# Modelling the spatial distribution of aquatic insects (Order Hemiptera) potentially involved in the transmission of *Mycobacterium ulcerans* in Africa

**DOI:** 10.1186/s13071-018-3066-3

**Published:** 2018-09-06

**Authors:** Jorge Cano, Antonio Rodríguez, Hope Simpson, Earnest N. Tabah, Jose F. Gómez, Rachel L. Pullan

**Affiliations:** 1 Faculty of Infectious & Tropical Diseases, London School of Hygiene & Tropical Medicine, London, UK; 2 Department of Horticulture, Botany and Landscaping, School of Agriculture, Food and Forestry Science and Engineering, University of Lleida, Lleida, Spain; 3 National Yaws, Leishmaniasis, Leprosy and Buruli ulcer Control Programme, Ministry of Public Health, Yaounde, Cameroon; 4 Department of Biodiversity, Ecology & Evolution, Complutense University, Madrid, Spain

**Keywords:** Aquatic insects, Order Hemiptera, *Mycobacterium ulcerans*, Buruli ulcer, ENFA, Ecological niche modelling

## Abstract

**Background:**

Biting aquatic insects belonging to the order Hemiptera have been suggested as potential vectors of *Mycobacterium ulcerans* in endemic areas for Buruli ulcer (BU). If this is the case, these insects would be expected to co-exist with *M. ulcerans* in the same geographical areas. Here, we studied the geographical distribution of six aquatic Hemiptera families that are thought to be vectors of *M. ulcerans* and explored their potential geographical overlapping with communities reporting BU cases in endemic countries.

**Methods:**

We have developed ensemble ecological models of predicted distribution for six families of the Hemiptera (Naucoridae, Belostomatidae, Notonectidae, Nepidae, Corixidae and Gerridae) applying a robust modelling framework over a collection of recorded presences and a suite of environmental and topographical factors. Ecological niche factor analysis (ENFA) was first used to identify factors that best described the ecological niches for each hemipteran family. Finally, we explored the potential geographical co-occurrence of these insects and BU in two endemic countries, Cameroon and Ghana.

**Results:**

Species of the families Naucoridae and Belostomatidae, according to our models, are widely distributed across Africa, although absent from drier and hotter areas. The other two families of biting Hemiptera, the Notonectidae and Nepidae, would have a more restricted distribution, being more predominant in western and southern Africa. All these four families of biting water bugs are widely distributed across coastal areas of West Africa. They would thrive in areas where annual mean temperature varies between 15–22 °C, with moderate annual precipitation (i.e. 350–1000 mm/annual) and near to water courses. Species of all hemipteran families show preference for human-made environments such as agricultural landscapes and urbanized areas. Finally, our analysis suggests that *M. ulcerans* and species of these aquatic insects might coexist in the same ecological niches, although there would be variation in species diversity between BU endemic areas.

**Conclusions:**

Our findings predict the geographical co-existence of some species of aquatic hemipteran families and BU. Considering the existing biological evidence that points to some of these aquatic insects as potential phoretic vectors of *M. ulcerans*, its presence in BU endemic areas should be considered a risk factor. The ecological models here presented may be helpful to inform future environmental based models intended to delineate the potential geographical distribution of BU in the African region.

**Electronic supplementary material:**

The online version of this article (10.1186/s13071-018-3066-3) contains supplementary material, which is available to authorized users.

## Background

Buruli ulcer (BU) is a chronic debilitating skin and soft tissue disorder caused by a bacterial infection, *Mycobacterium ulcerans*. A rapidly emerging disease in West Africa, BU is the third most common mycobacterial disease worldwide, after tuberculosis and leprosy. It is known to be endemic in at least 33 countries, but only 15 regularly report to the World Health Organisation (WHO) [1, 2]. Between 5000 and 6000 cases are reported annually by these countries, but despite a 2004 World Health Assembly resolution calling for increased surveillance and control, its true distribution and burden remain poorly understood [3]. This information is vital for planning and targeting appropriate control measures.

BU is a disease of rural areas, often escaping detection by national surveillance programs [4, 5]. This creates challenges for mapping the distribution of disease, and quantifying populations at risk. Although the exact mode of transmission for *M. ulcerans* has yet to be elucidated, the primary risk factor appears to be proximity to slow flowing or stagnant water [4]. Two potential pathways for an individual to become infected with *M. ulcerans* have been described: ingestion or inhalation of aerosolized bacteria and penetration through skin lesions by a contaminated environment (e.g. soil, water, vegetation, insect vector) [6]. Some lines of evidence suggest that biting aquatic insects (order Hemiptera) living in pools and streams may act as phoretic vectors (carriers) of *M. ulcerans* [7, 8], although other potential pathways such as mosquitoes [9, 10], amoebas [11] and even small mammals (wildlife) [12–14] have also been suggested. Whatever biotic or abiotic vehicle *M. ulcerans* uses to infect humans, it is clear that certain environmental settings, mostly influenced by human activity such as agriculture and rural settlements [15], and proximity to stagnant water are more favourable for the presence of *M. ulcerans* in endemic areas [16].

Assuming that the transmission of *M. ulcerans* might occur through different pathways [17], species of some families of aquatic Hemiptera such as the Belostomatidae, Naucoridae and Corixidae have been identified as potential vectors, or at least reservoirs, of the mycobacterial infection in marshy ecosystems from Ghana [18], Benin [19] and Cameroon [8, 20, 21]. Aquatic hemipterans are not hematophagous insects, do not need blood to develop any stage of their life-cycle, so that it is quite likely that humans become accidentally bitten by these insects because a purely defensive reaction or a simply predaceous instinct; some species show a very voracious and aggressive behaviour [22]. *Mycobacterium ulcerans* DNA has repeatedly been isolated from trapped samples of these insects, and the organism has been cultured from a species of the Gerridae caught in Benin [19]. Furthermore, experimentally infected species of the Naucoridae have been shown to be capable of transmitting *M. ulcerans* to a mouse model, which subsequently developed pathological signs consistent with Buruli ulcer [23]. Recent modelling work has also suggested a positive association between environmental suitability for insects of the Naucoridae and Belostomatidae and BU prevalence in Cameroon [24].

Regardless of whether aquatic Hemiptera act as vectors or reservoirs, what seems to be clear is that these aquatic insects and *M. ulcerans* may be sharing ecological niches in BU-endemic areas. Therefore, although their mere presence in the aquatic environment is insufficient to presume the transmission of *M. ulcerans*, it has been suggested that, in areas where BU cases are reported, they should be considered a potential risk factor along with many others biotic and abiotic factors [8].

Despite the evidence linking biting aquatic Hemiptera species and BU, little is known about the geographical distribution of these insects in Africa and their co-existence with BU. Here we present the first African-wide models delineating the potential geographical distribution of six aquatic Hemiptera families for which *in vivo* or ecological evidence has suggested a role in *M. ulcerans* transmission: Naucoridae, Belostomatidae, Notonectidae, Nepidae, Corixidae and Gerridae. We then explored the potential geographical overlap between BU and these hemipteran families in two endemic countries, Cameroon and Ghana.

## Methods

### Distribution of aquatic hemipteran families

In this study, we focused on the families Naucoridae, Belostomatidae, Notonectidae, Nepidae, Corixidae and Gerridae. The first four are considered biting insects, and thereby more likely to be involved in the transmission of *M. ulcerans* [8, 20]. Species of the Corixidae and Gerridae, although they are non-biting insects, have been found carrying *M. ulcerans*, thereby being attributed some role in the transmission of this bacteria [7, 19]. We retrieved georeferenced presence data for these hemipteran families from the Global Biodiversity Information Facility Database (GBIF) [25]. Occurrence data was cross-checked for spatial consistency (i.e. to verify that occurrence sites were correctly mapped according to country and location name) and remapped when necessary using OpenCage geocoder [26] and Google Engine Map. Geographical correction was done using the *ggmap* and *opencage* packages in R v.3.3.2.

Due to the scarcity of occurrence data for some of the Hemiptera families at the GBIF repository, systematic structured searches were conducted in electronic databases PubMed and Web of Science. We mostly gathered records of insect presence from research studies intended to identify risk factors associated with BU, and zoological catalogues (Table 1). When geographical coordinates of sampling sites were not provided by the source, the aforementioned R packages were used to obtain the precise location. Positioning was double-checked afterwards by mapping occurrence sites in Google Earth and confirming their reliability based on country and location name.

**Table 1:** Sources of presence data for the aquatic hemipteran families modelled, and the number of recorded locations in which traces of *M. ulcerans* DNA were detected on insects collected

Family	Source	No. of presences	Detected *M. ulcerans*			Reference
			na	No	Yes	
Naucoridae	GBIF	189	189			[74–80]
	Literature	250	245	2	3	
Belostomatidae	GBIF	190	190			[7, 22, 74, 77, 79, 80]
	Literature	43	37	1	5	
Notonectidae	GBIF	204	204			[7, 74, 77–79, 81]
	Literature	265	262	2	1	
Nepidae	GBIF	144	144			[74, 77–80]
	Literature	90	88	1	1	
Gerridae^a^	GBIF	138	138			[74, 79, 80]
	Literature	96	96			
Corixidae^a^	GBIF	294	294			[7, 74, 77–79]
	Literature	314	312		2	

*Abbreviation*: *na* not available or not investigated

^a^No biting species within the family

### Environmental datasets used in ecological modelling

A suite of environmental factors was considered to describe the ecological niche of each hemipteran family, and subsequently used to model their potential spatial distribution across Africa. Continuous gridded maps of climate, topography, vegetation and land use for Africa were obtained from different sources (Table 2). Various climate variables related to precipitation and temperature were downloaded from the WorldClim database [27]. The WorldClim database provides a set of global climate layers obtained by interpolation of precipitation data for the period 1950–2000 collected in weather stations distributed across the world [28]. Derived from the WorldClim datasets, the Consortium for Spatial Information (CGIAR-CSI) has produced gridded global estimates of aridity index and potential evapo-transpiration (PET) that are also freely available at 1 km^2^ resolution [29]. PET is a measure of the ability of the atmosphere to remove water through evapo-transpiration processes. Aridity is usually expressed as a generalized function of precipitation, temperature and/or PET. It can be used to quantify precipitation availability over atmospheric water demand. The global aridity index has been calculated dividing the mean annual precipitation by the mean annual potential evapo-transpiration (PET).

**Table 2:** Variables considered to characterize habitat of aquatic hemipteran families in Africa

Variable	Source
Annual cumulative precipitation^a^	WorldClim [27]
Maximum temperature	
Mean temperature^a^	
Minimum temperature	
Mean temperature of coldest quarter	
Mean temperature of warmest quarter	
Precipitation of driest quarter^a^	
Precipitation of wettest quarter	
Potential evapo-transpiration	CGIAR-CSI [29]
Aridity index	
Elevation^a^	
Slope^a^	Derived from elevation
Flow accumulation^a^	Derived from slope
Topographic wetness index^a^	Derived from slope and flow accumulation
Distance to rivers^a^	Digital Global Chart [32]
Distance to water-bodies^a^	Global Water Body Chart -WWF [31]
Land surface temperature (LST)^a^	AfSIS [36]
Enhanced vegetation index (EVI)	
Major land cover (forest, agriculture, shrubland-grassland)^a^	Global Land Cover 2000 [34]

^a^Environmental predictors which were finally selected for ecological niche modelling after checking for potential collinearity (correlation coefficient ≥ 0.8)

Also from CGIAR-CSI, we obtained a raster dataset of elevation at 1 km^2^. This elevation layer resulted from processing and resampling the gridded digital elevation models (DEM) derived from the original 30-arcsecond DEM produced by the Shuttle Radar Topography Mission (SRTM). We used the elevation layer to generate two topography-related datasets: slope of terrain and flow accumulation. Slope was obtained in degrees.

To produce the flow accumulation layer, we initially created a flow direction layer, in which the direction of flow was determined by the direction of the steepest descent, or maximum drop, from each cell in the elevation dataset. This was calculated as follows: change in elevation value / distance * 100. Flow accumulation was then calculated as the accumulated weight of all cells flowing into each downslope cell in the flow direction layer.

In addition, we calculated the topographic wetness index by applying the following algorithm

$$ TWI=\ln \left(a/ tan\beta \right) $$

where *a* is the upslope contributing area per unit contour length (or Specific Catchment Area, SCA), which can be approached by using the flow accumulation, and *β* is the local slope gradient for reflecting the local drainage potential [30].

We also produced continuous surfaces of straight line distance (Euclidean distance) in km to the nearest water body and permanent rivers based on the Global Database of Lakes, Reservoirs and Wetlands [31] and Digital Global Chart [32] respectively.

Land cover data were downloaded from the GlobCover project at the European Space Agency [33, 34]. This global land cover map is derived by an automatic and regionally-tuned classification of a 300-m MERIS FR time series (19 months) and comprises 22 land cover classes according to the UN Land Cover Classification System (LCCS) [35]. We simplified this raster dataset by grouping the 22 land cover classes into 4 major groups; agricultural land (crops), grassland-shrubland, forest areas and woodlands, and others (i.e. bare soil, urbanized areas and snow/rock).

Finally, raster datasets of averaged Enhanced Vegetation Index (EVI) and land surface temperature (LST) for the period 2000–2015 were obtained from the African Soil Information System (AfSIS) project [36]. This project generates time series average products for several environmental indicators such as vegetation indices and LST using MODIS satellite image data collected by the National Aeronautics and Space Administration (NASA). The MOD13Q1 product, which is updated every 16 days at 250m spatial resolution, includes vegetation indices such as Normalized Difference Vegetation Index (NDVI) and EVI [37]. Compared to the NDVI, the EVI minimizes canopy background variations (which can lead to errors in vegetation classification), and maintains greater sensitivity over dense vegetation conditions. Day and night LST data are generated from MOD11A2 products, and have a spatial and temporal resolution of 1 km and 8 days, respectively [38].

Input grids were resampled to a common spatial resolution of 5 × 5 km using nearest neighbour approach, clipped to match the geographic extent of a map of mainland Africa, and eventually aligned to it. Raster manipulation and processing was undertaken using *raster* package in R v.3.3.2. Final map layouts were created with ArcGIS 10.3 software (ESRI Inc., Redlands CA, USA). Only non-linearly related covariates were considered for subsequent analysis. This variable selection was done by implementing a stepwise procedure to identify a set of non-correlated variables that will have low variance inflation factor (VIF). VIF is calculated as follows: ${VIF}_i=1/1-{R}_i^2$ , where ${R}_i^2$ is the coefficient of determination of the regression for each variable as a function of all remaining predictors. We retained a variable combination which resulted in a VIF below 10.

### Ecological Niche Factor Analysis

As a first step to build the ecological niche models, an Ecological Niche Factor Analysis (ENFA) [39] was performed with Biomapper 4 [40]. ENFA analysis is widely used to explore habitat suitability and compute species distribution maps without absence data [39, 41]. This analysis was used to identify predictors most likely to limit the insects’ distribution, and for reducing dimensionality, thereby avoiding the inclusion of several correlated variables [42]. The output of this analysis includes non-correlated factors, similar to that obtained by a Principal Components Analysis (PCA), with the particularity that factors obtained always have ecological significance [39].

Employing the environmental variables described above as predictors a set of factors for each of the Hemiptera families were obtained. Factors were selected by the broken stick criterion: they were retained if they explained larger variance than the broken stick null model, which assumes that the variance has been divided at random among the factors [43]. This method is only appropriate for continuous data, and thus land cover classification was excluded for this stage.

The marginality factor (MF), the specialization factors (SF), and global marginality and tolerance values of each species were considered to support the interpretation of the niche models. MF is the first component obtained by ENFA and describes how far the family optimum niche is from the mean habitat in the study area, for each predictor. SF are extracted successively from the *n*-1 residual dimensions. High absolute factor loadings of a variable on SF indicate a more restricted range of the family on the corresponding variable. The global marginality takes account of the MF scores of all the predictors and gives a summary of how much the family habitat differs from the available average conditions. A low value (close to 0) indicates that the family tends to live in average conditions throughout the study area. A high value (close to 1) indicates a tendency to live in the most extreme conditions of the study area. The global tolerance value considers the eigenvalues of all the factors obtained by ENFA and indicates how specialized the family niche is in relation to the overall model area. It can have values between 0 and 1, and the higher is it, the wider is the range of environmental conditions that the species bears. Thus, a value close to 0 indicates a specialist taxon and a value close to 1 indicates a generalist one [39].

For each hemipteran family, variables with a factor loading higher than 0.3 were considered to best define the insects’ ecological niche [41]. Variables with a factor loading above this cut-off were retained to create sets of environmental factors that were used for the distribution modelling of each family.

### Developing ecological models

An ensemble of distribution models was generated for each hemipteran family, based on the presence data collected and the environmental factors identified through ENFA analysis. We used seven algorithms available within the BIOMOD framework [44] to obtain those ensembles of predicted distributions: generalized linear models (GLM), generalized additive models (GAM), generalized boosted regression models (GBM), artificial neural networks (ANN), multiple adaptive regression splines (MARS), maximum entropy (MaxEnt) and random forest (RF). These models were run using the parameters set by default in the *biomod2* R package [44], except for the GBM models. As per guidelines from Elith et al. [45], the learning rate (*lr)* and tree complexity (*tc*), key parameters in GBM models, were set according to the number of observations and testing different values on a subset of samples (75%), using deviance reduction as the measure of success. Overall, *lr* of 0.005 and *tc* of 5 were identified as optimal parameters, thereby enabling the model to account for up to 5 potential interactions and slowing it down enough to get the model converged without over-fitting the data. This tuning was undertaken using the *gbm* package in R v.3.3.2.

All these models are intended to discriminate the suitability of the environment for the presence of a particular organism, and for this they need to be trained with presence and absence records. When there are no available absences records, an alternative is to generate background points or pseudo-absences [46]. We generated sets of the same number of background points as presence data compiled for every hemipteran family. Background points were randomly generated accounting for the underlying geographical bias on the occurrence data, as previously recommended [47]. For this, we created a sampling bias surface by counting the number of occurrence records within each grid cell (5 × 5 km resolution) and then extrapolated these data across Africa using kernel density estimation using the R packages *kernlab*, *ks* and *sm*. Lastly, we generated the background points from random locations weighted by the sampling bias surface [48, 49].

Models were calibrated using an 80% random sample of the initial data and evaluated against the remaining 20% data using the area under the curve (AUC) of the receiver operation characteristic (ROC) and the true skill statistic (TSS) [50]. Projections were performed 80 times, each time selecting a different 80% random sample while verifying model accuracy against the remaining 20%. Verification or internal evaluation does not allow for assessment of the predictive performance of the models - independent evaluation data would be required for this purpose - but it provides a measure of internal consistency of the models. Unfortunately, the scarcity of occurrence records collected did not allow for independent cross-validation of the final models. The evaluation statistics (AUC and TSS) were used to select the models to be assembled on the basis of matching between predictions and observations. Here, models with AUC < 0.8 or TSS values < 0.7 were disregarded when constructing the final assemble model.

The final assemble model was obtained by estimating the median of probabilities across the selected models per Hemiptera family and per grid cell. This approach was used alternatively to the mean because is less sensitive to outliers [51]. The range of uncertainties obtained with the seven modelling techniques was also calculated by estimating the confidence intervals across the ensemble for each hemipteran family and per grid cell.

### Data sources on Buruli ulcer occurrence

Two difference datasets were used to explore the potential geographical overlap between BU occurrence and the predicted distribution of aquatic Hemiptera insects. A data table with a list of 91 georeferenced villages from southwestern Ghana that reported BU cases during 2007–2010 was obtained from a recent publication [52]. A second dataset was provided by the National Committee for Yaws, Leishmaniosis, Leprosy and Buruli ulcer Control in Cameroon (Cameroon Ministry of Health) and included a list of 414 communities that reported BU cases during 2003–2015. We decided to retain for this analysis those communities providing accurate geographical coordinates and reporting PCR-confirmed BU cases in early stages of disease (to exclude potentially imported cases). A map displaying the georeferenced locations from Cameroon and Ghana has been included in a supplementary file (Additional file 1: Figure S1).

## Results

### General description of datasets

A total of 2217 records of occurrence were compiled for the six hemipteran families targeted in this work: 439 for the Naucoridae (Additional file 2: Table S1); 233 for the Belostomatidae (Additional file 2: Table S2); 469 for the Notonectidae (Additional file 2: Table S3); 234 for the Nepidae (Additional file 2: Table S4); 234 for the Gerridae (Additional file 2: Table S5); and 608 for the Corixidae (Additional file 2: Table S6). Based on the source of data, 52.3% (1589/2217) were extracted from the GBIF and 47.7% (1058/2217) through systematic literature searches (Table 1). The proportion of occurrences by source varies across the families, as shown in Table 1. Only 18 recorded occurrences were associated with BU studies and in 10 of them *M. ulcerans* was found (by PCR test) within studied specimens.

**Fig. 1:**
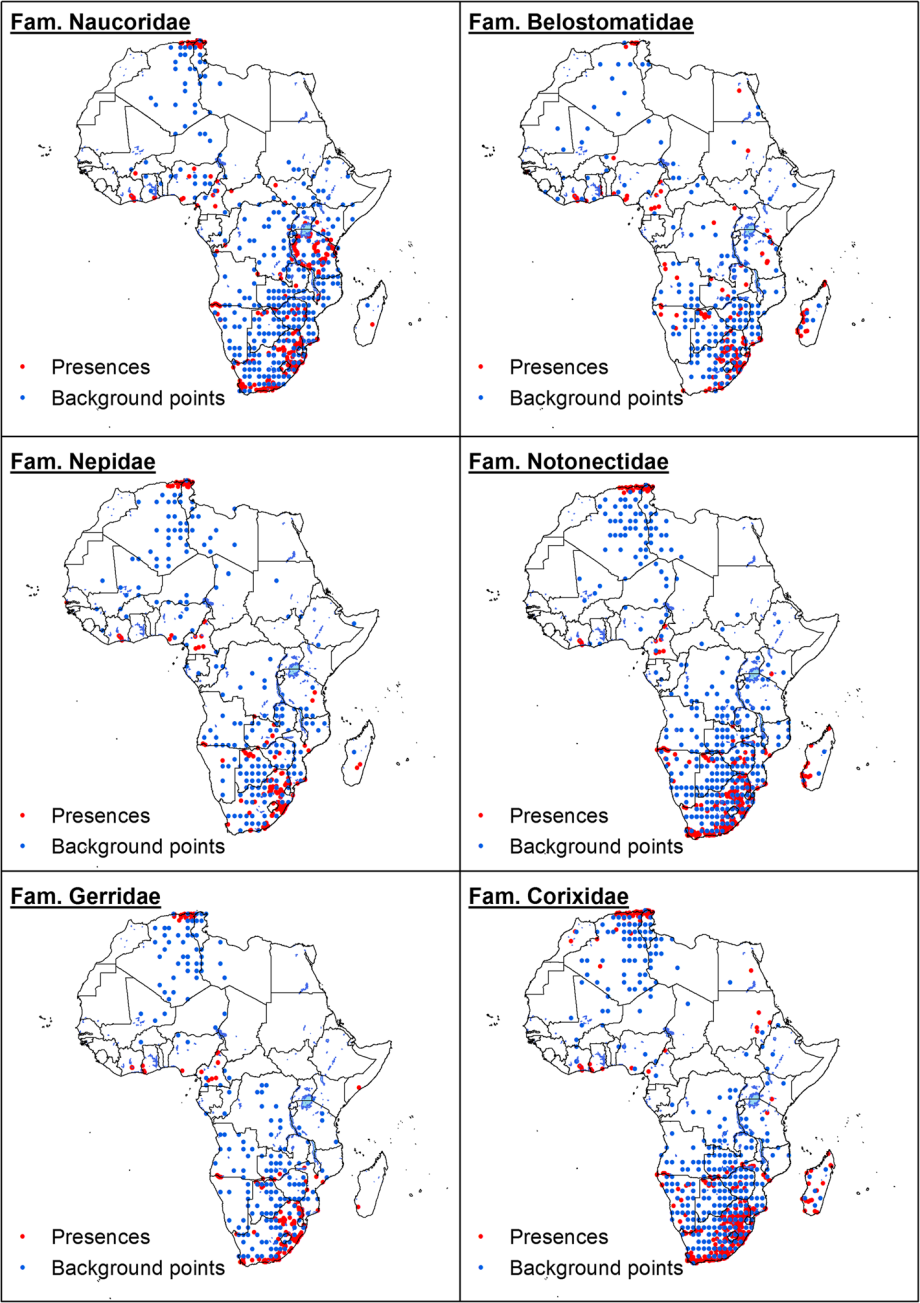
Distribution of presence data and background points for the aquatic hemipteran families studied

Most of the collected occurrences are distributed across southern, western and northern Africa, although geographical distribution varies between families (Fig. 1). Naucoridae and Belostomatidae occurrences are geographically more widespread, extending to eastern Africa. For others, such as the Gerridae and Corixidae, recorded occurrences are restricted to northern and southern Africa. Figure 1 also shows the distribution of background points (“pseudo-absences”) which, as detailed in method section, were generated to account for the geographical bias in the distribution of occurrence records.

### ENFA analysis

ENFA identified the most relevant predictors for each family and provided some insights on their ecological niches. Marginality factor values for the six families (Table 3) indicate that they are each sensitive to temperature variables and associated with areas that are colder than the mean across Africa (in terms of mean temperature, long term LST and temperature in the warmest quarter). The Belostomatidae and Naucoridae were both predicted to be present in areas where average temperature in the coldest quarter is close to the mean across Africa, whereas the other families were associated with areas with a lower mean temperature in the coldest quarter. The Naucoridae was the only family found to be strongly influenced by slope and favored by habitats with steeper gradients.

**Table 3:** Marginality factor (MF) scores and global marginality index for selected aquatic hemipteran families in Africa

Environmental variable	Family					
	Naucoridae	Belostomidae	Notonectidae	Nepidae	Gerridae	Corixidae
Annual precipitation	0.157	0.211	0.04	0.106	0.092	0.038
Mean temperature	-0.486^a^	-0.456^a^	-0.578^a^	-0.558^a^	-0.539^a^	-0.586^a^
Long-term mean LST	-0.417^a^	-0.471^a^	-0.373^a^	-0.453^a^	-0.442^a^	-0.36^a^
Precipitation of driest quarter	0.22	0.212	0.156	0.142	0.194	0.125
Euclidean distance to water bodies in km	-0.117	-0.235	-0.086	-0.124	-0.093	-0.088
Euclidean distance to rivers in km	-0.171	-0.241	-0.157	-0.182	-0.175	-0.159
Flow accumulation	0.027	0.073	0.017	0.018	-0.002	0.026
Temperature of coldest quarter	-0.275	-0.265	-0.435^a^	-0.42^a^	-0.389^a^	-0.45^a^
Temperature of warmest quarter	-0.484^a^	-0.508^a^	-0.468^a^	-0.452^a^	-0.457^a^	-0.464^a^
Slope	0.367^a^	0.171	0.221	0.128	0.244	0.196
Elevation	0.083	0.062	0.065	0.065	0.003	0.124
Wettest index	-0.148	0.008	-0.084	-0.04	-0.095	-0.069
Explained specialization (%)	42.8	46.8	34	52.5	55.9	36.9
Global marginality	1.172	0.869	1.271	1.179	1.233	1.287

^a^Variables with an absolute value > 0.3 are considered highly influential

The Belostomatidae scored the lowest value for the global marginality index (Table 3), indicating that this family would be most likely to occupy average or normal habitats, according to the analyzed environmental predictors, throughout Africa (Table 3).

Considering all the factors obtained by ENFA, we were able to select the most important variables associated with each family’s distribution (Table 4). Besides the variables mentioned above, distance to rivers was also found to drive the distribution of the six aquatic Hemiptera, but distance to water bodies was not found to be relevant.

**Table 4:** Variables selected through ENFA for each aquatic hemipteran family (MF or SF scores > 0.3)

Environmental variable	Family					
	Naucoridae	Belostomidae	Notonectidae	Nepidae	Gerridae	Corixidae
Annual precipitation			**•**			
Mean temperature	**•**	**•**	**•**	**•**	**•**	**•**
Long-term mean LST	**•**	**•**	**•**	**•**	**•**	**•**
Precipitation of driest quarter				**•**		
Euclidean distance to rivers in km	**•**	**•**	**•**	**•**	**•**	**•**
Flow accumulation			**•**	**•**	**•**	**•**
Temperature of coldest quarter			**•**	**•**	**•**	**•**
Temperature of warmest quarter	**•**	**•**	**•**	**•**	**•**	**•**
Slope	**•**					
Wettest index			**•**		**•**	**•**
Number of factors	2	2	4	4	4	4
Global tolerance	0.422	0.411	0.41	0.337	0.306	0.437

Flow accumulation was predicted to condition the distribution of the families Corixidae, Gerridae, Nepidae and Notonectidae. Considering the global tolerance index (Table 4), the Gerridae and Nepidae seem to have more restricted ecological niches in Africa than the rest of the hemipteran families.

### Environmental suitability

A suite of 560 ensembles obtained by different modelling approaches (GLM, GAM, GBM, RF, ANN, MARS and MaxEnt) - 80 per each - were run with a random sample of 80% of the total occurrences and background points for each hemipteran family. Only those ensembles with a high predictive performance (TSS > 0.7 or AUC > 0.8) contributed to the final model. Additional file 1: Table S1 shows the median value of statistic indicators and 95% confidence interval (95% CI) of their distribution. Boosted regression trees (GBM) and random forest (RF) consistently outperformed the other modelling approaches, thereby primarily contributing to the final ensemble model. However, a number of other models also informed the final predictive models, as can be inferred from their TSS and AUC values (Additional file 1: Table S1).

**Fig. 2:**
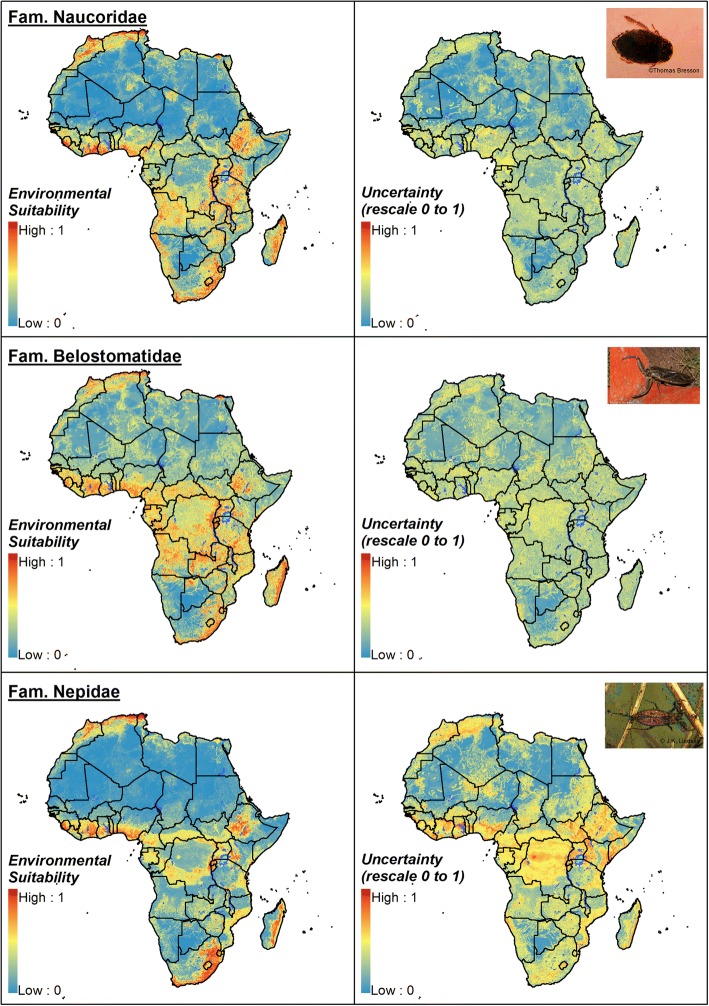
Ensemble consensus model for the hemipteran families Naucoridae, Belostomatidae and Nepidae: environmental suitability and uncertainty of prediction. Uncertainty was calculated as the range of the 95% confidence interval in predicted probability of occurrence for each pixel and rescaling to a 0–1 scale. Insect images from Wikimedia Commons (Wikipedia Public domain image resources)

**Fig. 3:**
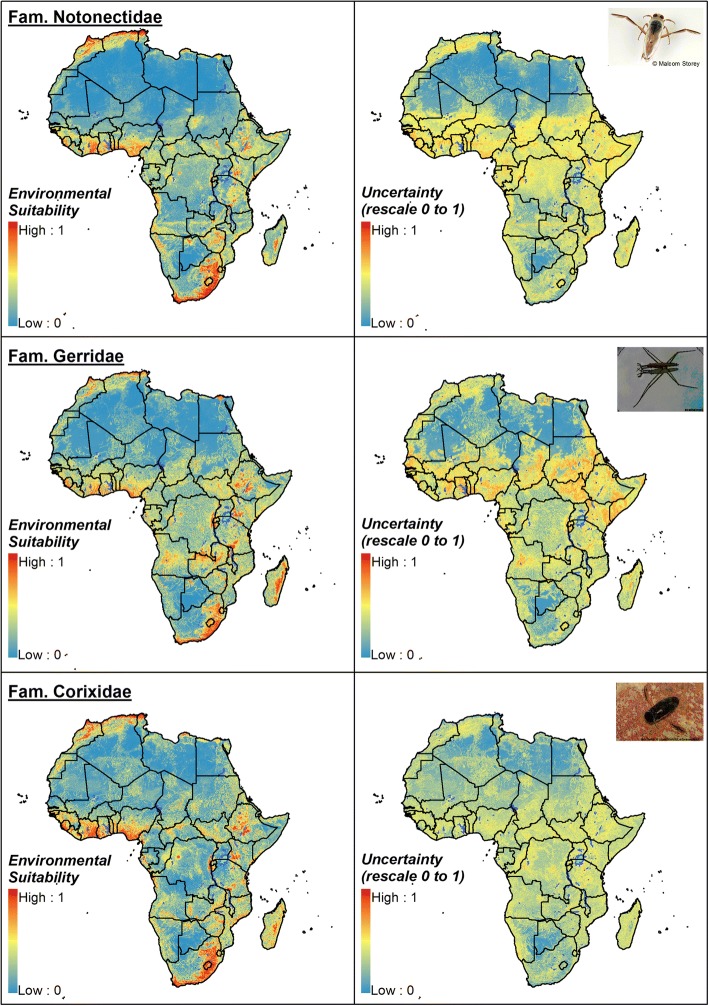
Ensemble consensus model for the hemipteran families Notonectidae, Gerridae and Corixidae: environmental suitability and uncertainty of prediction. Uncertainty was calculated as the range of the 95% confidence interval in predicted probability of occurrence for each pixel and rescaling to a 0–1 scale. Insect images from Wikimedia Commons (Wikipedia Public domain image resources)

Figures 2 and 3 display the environmental suitability for the presence of each hemipteran family across Africa and the uncertainty of prediction, this being calculated as the range of the 95% confidence interval in predicted probability of occurrence for each pixel and rescaling to a 0–1 scale. Coastal areas of West Africa, and the south-east of South Africa were predicted to be highly or moderately suitable for all families, and parts of Central Africa and East Africa were suitable for all biting families. Predicted suitability for the families Nepidae, Notonectidae and Gerridae in endemic areas of West and Central Africa is associated with high uncertainty, most likely associated with a lower number of presences recorded in these regions, but suitability for the Naucoridae, Belostomatidae and Corixidae is accompanied by moderate levels of uncertainty. The Belostomatidae was predicted to have the widest suitability range. The Sahara was predicted to be generally unsuitable for all families, but with pockets of moderate suitability for the Naucoridae and Belostomatidae. These predictions were associated with high certainty. Additional file 3: Figures S1-S24 provide more details on findings from ecological modelling for each hemipteran family: (i) maps of predicted suitability (median probability of occurrence) and 95% CI lower and upper bound maps; (ii) binary maps displaying the areas where their occurrence is more likely based on optimal cut-off; and (iii) a detailed description of marginal effect plots for GBM and RF models.

In summary, the families Naucoridae and Belostomatidae show a ubiquitous geographical distribution across Africa, except throughout drier and hotter areas such as the Kalahari and Sahara deserts. The other two families of biting Hemiptera, the Notonectidae and Nepidae, would have a more restricted distribution, being more predominant in western and southern Africa and narrowly present in middle Africa. All these four families are widely distributed across coastal areas of West Africa, although the Belostomatidae distribution would extend towards more inland ecosystems of western African countries. Of the hemipteran families considered non-biting, the Corixidae would be spread across Africa, even in desert areas from Sahel, whilst the Gerridae, a well-known cosmopolitan hemipteran family, would have a more limited distribution, according to our environmental models.

Additional file 1: Figures S2-S3 show the relative contribution (average) of selected environmental variables to the final predictive models. Although there are differences between families, land surface temperature appeared to be the main factor driving the distribution of all hemipteran families, followed by other temperature-related parameters such as mean temperature in the warmest and coldest quarters. According to the marginal effect plots (Additional file 3: Figures S3, S4, S7, S8, S11, S12, S15, S16, S19, S20, S23, S24), all these aquatic Hemiptera seem to thrive under mean temperature between 15–22 °C and near to water courses. However, the suitability for their presence declines sharply when LST exceeds 30 °C. The family Naucoridae, however, seems to tolerate even higher temperature according to their marginal effect plots (Additional file 3: Figures S3 and S4). In general, optimal ecological niches for these hemipteran families are characterized by moderate annual average precipitation (i.e. 350–1000 mm/year). The four biting Hemiptera families show preference for transformed ecosystems such as agricultural landscape and human settlements over forest or grasslands.

**Fig. 4:**
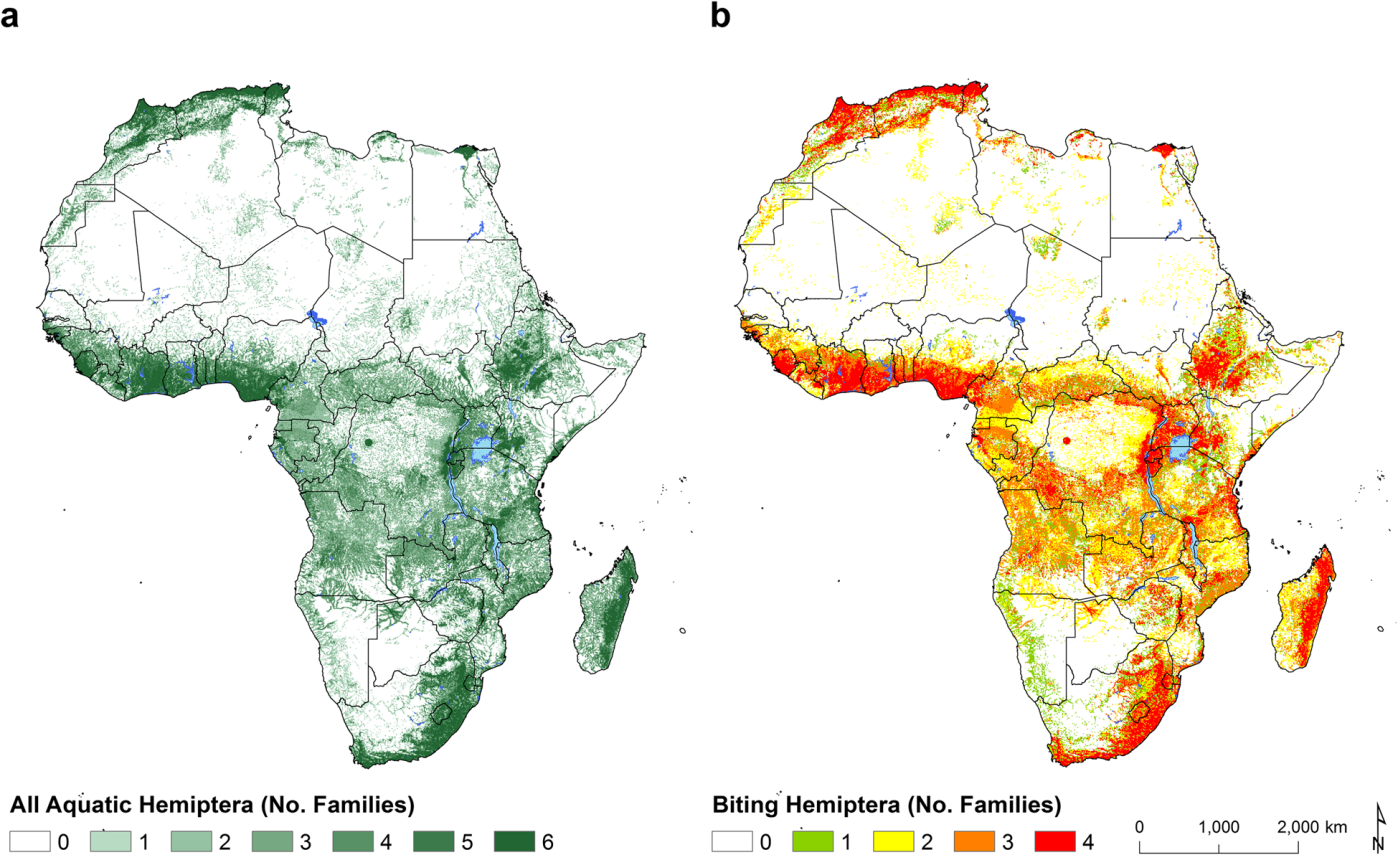
Predicted overlapping of aquatic hemipteran families, all (**a**) and biting families (**b**), across Africa. Maps shows number of families potentially present by 100-km^2^ area. The Naucoridae, Notonectidae, Belostomatidae and Nepidae are considered potentially biting insects

Figure 4 shows the potential for concurrent geographical distribution of all these hemipteran families. Our models predict the geographical overlapping of the biting Hemiptera families in broad areas of western Africa, southern and northern Africa, highlands of Ethiopia and Uganda, the northernmost and south-west coasts of the continent, the east coast of Madagascar and in pockets across eastern Africa.

### Geographical overlap with Buruli ulcer

**Fig. 5:**
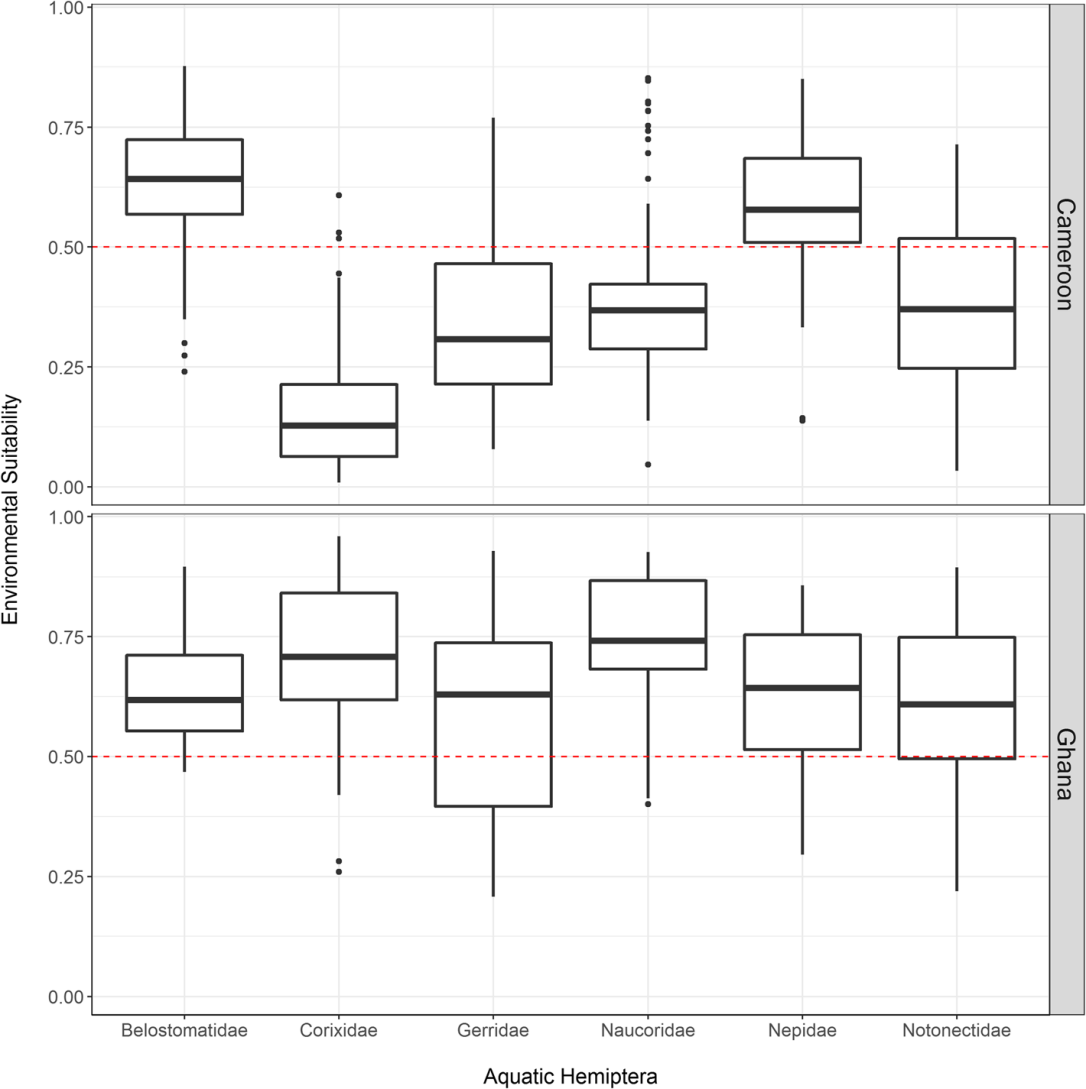
Predicted presence of aquatic hemipteran families in Buruli ulcer-endemic areas of Cameroon and Ghana. Dashed red line is the averaged optimal threshold above which presence of aquatic hemipteran insects is more likely

**Fig. 6:**
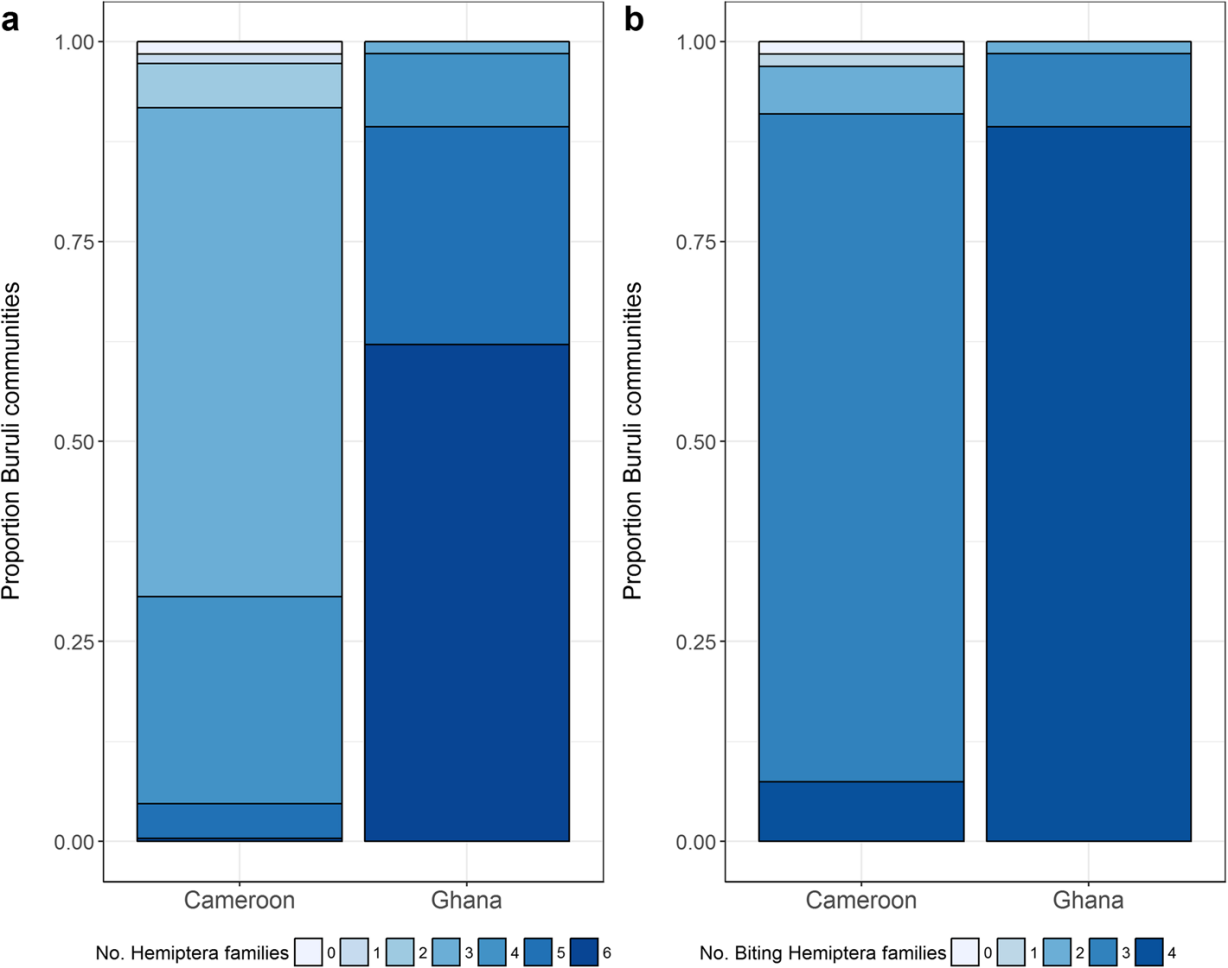
Diversity of aquatic hemipteran families, all (**a**) and biting families (**b**), potentially coexisting in Buruli ulcer-endemic areas of Cameroon and Ghana. Maps shows number of families potentially present by 10 × 10 km area

A simple exploratory analysis overlaying geolocated communities that have reported BU cases and our predictive models shows that *M. ulcerans* and species of these aquatic hemipteran families might coexist in the same ecosystems (Fig. 5, Additional file 1: Figure S1). Although all BU-endemic areas in both countries were suitable for at least one hemipteran family, our analysis suggests a marked heterogeneity in this relationship: BU-endemic areas in Cameroon were only suitable for two of the six hemipteran families, whereas BU-endemic areas in Ghana were predicted to be suitable for all six families (Fig. 5). There also seems to be spatial heterogeneity in the diversity of hemipteran families present in BU foci from West and Central Africa. Over 75% of BU-endemic communities from Ghana here studied would have the environmental conditions for five out of the six hemipteran families to prosper, while in Cameroon, less than 10% communities of the BU-endemic communities would have suitable conditions for 4 of the hemipteran families. This difference is even more significant when we restrict the analysis to biting insects (Fig. 6).

## Discussion

Here we present the first African-wide predictive distribution maps for aquatic hemipteran families potentially associated with the transmission of *M. ulcerans* in areas endemic for the disabling neglected tropical disease BU. Our findings point to potential geographical co-existence of these aquatic insects and BU foci from Cameroon and Ghana, which might extend to other BU-endemic countries in Africa. It is nonetheless worth mentioning that although the modelling approach used to construct these models was intended to minimize the uncertainty by assembling multiple models that provide the best guess of their potential distribution (model selection limited by certain predetermined threshold on cross-validation statistics), this will always exist particularly for areas that are little informed (few presences recorded). Thus, we must recognize that the uncertainty is significant for some families in areas from West and Central Africa, what may introduce some variation on the probability of occurrence within the BU-endemic areas analysed here.

From the maps, it is evident that these aquatic insects exhibit a broader distribution than the disease [3, 53], which is mostly confined to West and Central Africa, and has never been reported from any of the north-eastern or south-western African countries where we identified suitability for several of the hemipteran families. Insect vector-borne diseases commonly have a more restricted range than the organisms that carry them because transmission also relies on the presence of the infectious agent in the environment, which is influenced by many other biotic and abiotic factors [54]. A recent work has presented a model of the potential distribution of the Naucoridae and Belotomatidae insects in Cameroon based on a set of six environmental variables and records of presence of these insects in 36 sample sites across the country [24]. The authors also found a positive correlation between the environmental suitability for the presence of Naucoridae and Belostomatidae insects and BU prevalence, although they restrained from making any causative assumption.

Aquatic macroinvertebrates were first identified as potential vectors of *M. ulcerans* by Portaels et al. [55], who suggested *M. ulcerans* may be moved between trophic levels and feeding groups within the aquatic community. A more recent investigation has come to reinforce this hypothesis, demonstrating the transmission of *M. ulcerans* to specimens of the Belostomatidae that were fed on infected mosquito larvae [56]. This would explain why *M. ulcerans* has been found in a wide range of aquatic organisms, aside from aquatic biting insects, such as snails, plants and other aquatic macroinvertebrates [57–60]. The role of aquatic biting insects in the transmission of *M. ulcerans* has been supported by laboratory experiments, through the isolation and subsequent culture of *M. ulcerans* from saliva samples from a water strider (family Gerridae) [19] and by demonstrating its transmission from infected specimens of the Naucoridae biting a mammal model [22]. These various lines of evidence suggest that the most likely role for these bugs in the transmission of *M. ulcerans* is purely mechanical (i.e. as carriers or phoretic vectors), as *Musca* flies play in the transmission of *Chlamydia trachomatis*, the causative agent of trachoma [61]. However, it is yet to be elucidated how the insects become naturally infected (e.g. through contact with ulcers on human cases or preying on infected microinvertebrates) and whether the transmission, in both directions (environment-prey/ Hemiptera-human), is frequency-dependent or density-dependent. Nor has it been deeply studied whether some species may be more suitable vectors for *M. ulcerans* than others, according to their predatory habits and habitat preferences. For instance, the Belostomids, also colloquially knows as “toe-biters” or “alligator ticks”, are aggressive predators, capable of flying, and have been reported to bite humans. Thus, in Cameroon a BU infection in a child was reportedly attributed to a bite of a Belostomid insect near a water canal [22].

The order Hemiptera is broadly documented to inhabit a wide range of freshwater ecosystems worldwide, with different families displaying distinct habitat preferences [62–64]. Commonly, hemipteran communities are more diverse in warm, heavily vegetated, lentic or slow lotic waters with increased nutrient levels than in rivers. Nevertheless, in a recent study in Cameroon, aquatic hemipteran insects were found in a large variety of aquatic ecosystems such as lakes, flood areas and swamps, but were also present in surveyed streams and rivers [65]. This is consistent with our findings that show higher environmental suitability for all the hemipteran families in areas near to rivers and streams, and also areas prone to flooding: with low slope and high flow accumulation value (marginal effect plots in Additional file 2). Surprisingly, our gridded map displaying distance to water bodies was not selected by ENFA analysis to describe the ecological niches for any of the hemipteran families. This might be explained by the coarse spatial resolution of the only inland water map available. Thus, small water bodies, which may be more suitable for these aquatic insects, are not represented in this global map of lakes, reservoirs and wetlands [31].

The major types of water-bodies with records of hemipteran insects (small ponds/swamps, streams/rivers, and lakes) are all present in the coastal areas of West Africa, considered at the highest risk for BU. For lentic water-bodies (ponds and lakes), environmental suitability may depend on size. Lakes have higher environmental stability, number of niches, and probability of immigration of new species, whereas the smallest water bodies are shallow, and their small water volumes make them unstable environments, more susceptible to seasonal variations in their fauna composition [65–67]. Assuming a role of macroinvertebrate fauna in the transmission of *M. ulcerans*, we should in turn expect a seasonal oscillation in the presence of *M. ulcerans* in small pools. This has been proven in a thorough ecological study conducted in Cameroon, where authors observed monthly and rainfall-related variation in the *M. ulcerans* positivity rate of analysed pools and also among taxonomic orders of aquatic insect colonizing those pools [65]. This would ultimately explain the marked seasonal pattern of BU incidence in foci of Central [68] and West Africa [69].

There is a lack of previous contributions exploring distribution patterns of aquatic hemipteran insects and their association with large-scale environmental and climate related factors. The few that have been carried out, aligned with our findings, indicated a preference of the four biting hemipteran families studied here for transformed ecosystems such as agricultural landscapes and rural settlements over forest or grasslands [24]. Although this suggests that land cover type influences hemipteran distribution patterns, it has previously been argued that due to the migration and dispersal abilities of this insect group, the land use in wide-scale terms may not actually limit its distribution in practice [64].

The results of this study suggest the existence of three types of hemipteran assemblages according to their distribution patterns and habitat preferences: Naucoridae-Belostomatidae, Notonectidae-Nepidae and non-biting Corixidae-Gerridae families. Nevertheless, the fact of analysing hemipteran fauna as family groups may determine this spatial suitability pattern, as hemipterans regularly shared ecological traits within taxonomic groups [70, 71]. In this investigation, although our models suggest different geographical ranges for each assemblage, all of them were restricted to areas of moderate temperature and annual average precipitation, as has been found for other insect complexes. Inexplicably, marginal plots of temperature-related variables for the Naucoridae show two peaks of moderate suitability at extreme temperatures. Although this may be explained by a greater tolerance of this family to higher temperatures, it may also be attributed to an error in the geolocation of recorded occurrences.

Some previous contributions have defined particular ecological traits for these hemipteran families which may be related to the observed differences in their distributions [24, 72]. Gerridae and Nepidae seemed to be the least tolerant families, as deduced by the lower global tolerance index from the results of the ENFA analysis (Table 4). Gerridae is the only one of the six families whose species live and move across water surfaces. Their association with distance to rivers is less marked, and they show a stronger preference for environments with moderate flow accumulation, compared to the other families (Additional file 3: Text S6, Figures S23 and S24). This may suggest that Gerridae can inhabit small waterbodies, perhaps not captured in our environmental layers. On the other hand, the Nepidae are non-flying insects which live at the bottom of the water column. Consequently, it is not surprising that they are quite dependent on precipitation, especially during driest periods, as the variable contribution graph shows (Additional file 1: Figure S2), since they could be more restricted by summer droughts. Finally, the Naucoridae is the only family favoured by moderate slopes (Additional file 3: Text S1, Figures S3 and S4). Riverine vegetation located on moderate slopes may provide favourable habitat conditions which compensate their inability to fly. Microhabitat composition is of major importance to hemipteran assemblages and distribution, and the existence of large plants near the water-bodies provides appropriate habitat for the Naucoridae and Belostomatidae to forage, develop and reproduce [24, 73].

As wider distribution patterns by family taxa have been explored in this study, ecological factors concerning water composition, chemical and physical structure have not been analysed. These factors play a more important role in species distribution and their relative abundance in aquatic ecosystems at lower spatial scale, as some studies have shown [18]. It is well known that factors such as water temperature, composition of aquatic vegetation, sediment, calcium/magnesium concentration or water pH could determine local distribution of hemipteran species [64]. Unfortunately, we have been unable to develop species-specific distribution models due to the scarcity of available occurrence records for the species potentially involved in BU transmission. We must acknowledge this fact as a limitation of our study, which should be addressed in further research endeavours.

Existing evidence points to a mechanical role of these aquatic insects in the transmission of *M. ulcerans*, and in this capacity, we would expect there to be differences between species due to differences in predatory behaviour and habitat preferences. The actual contribution of aquatic Hemiptera in the transmission of *M. ulcerans* to humans in nature is still to be confirmed. If demonstrated, identifying the species actually involved and characterizing their ecological niches at micro-scale level might become relevant to understand the mechanism of this transmission pathway and improve disease surveillance in at-risk areas.

## Conclusions

To our knowledge, the maps presented in this work are the first attempt to model the environmental suitability for the presence of aquatic Hemiptera insects across Africa. We found broad suitability for the occurrence of these water bugs across much of the continent, especially in areas considered to be high risk for BU, but also in large areas from where BU has never been reported. We also identified variation in the type and diversity of species present in BU foci. The distribution models that we have developed for major Families of aquatic biting Hemiptera may contribute to construct future environmental-based models intended to delineate the potential geographical distribution of BU in the African region.

## Additional files

Additional file 1: **Table S1.** Median and 95% confidence intervals of validation indicators (TSS, AUC and PCC) for the modelling approaches tested: generalized linear models (GLM), generalized additive models (GAM), boosted regression trees (GBM), artificial neural networks (ANN), multiple adaptive regression splines (MARS), maximum entropy (MAXENT) and random forest (RF). **Figure S1.** Distribution of communities reporting Buruli ulcer in Ghana (2007–2010) and Cameroon (2003–2015) over a gridded map of predicted distribution of Hemiptera families. **Figure S2.** Variable contribution of final ensemble models based on GBM and RF algorithms for the Naucoridae, Belostomatidae and Notonectidae. **Figure S3.** Variable contribution of final ensemble models based on GBM and RF algorithms for the Nepidae, Corixidae and Gerridae. (DOCX 795 kb)

Additional file 2: **Table S1.** Recorded occurrences for the family Naucoridae. **Table S2.** Recorded occurrences for the Belostomatidae. **Table S3.** Recorded occurrences for the Notonectidae. **Table S4.** Recorded occurrences for the Nepidae. **Table S5.** Recorded occurrences for family Gerridae. **Table S6.** Recorded occurrences for family Corixidae. (XLSX 151 kb)

Additional file 3: **Figure S1.** Environmental suitability for the Naucoridae across Africa and prediction uncertainty (95% CI). **Figure S2.** Predicted occurrence for the Naucoridae across Africa and uncertainty. **Figure S3.** Partial dependence plots of the relative contribution of covariates to the boosted regression tree (BRT) model for the Naucoridae. **Figure S4.** Partial dependence plots of the relative contribution of covariates to the random forest (RF) model for the Naucoridae. **Text S1.** Description of ecological niche for the Naucoridae across Africa. **Figure S5.** Environmental suitability for the Belostomatidae across Africa and prediction uncertainty (95% CI). **Figure S6.** Predicted occurrence for the Belostomatidae across Africa and uncertainty. **Figure S7.** Partial dependence plots of the relative contribution of covariates to the boosted regression tree (BRT) model for the Belostomatidae. **Figure S8.** Partial dependence plots of the relative contribution of covariates to the random forest (RF) model for the Belostomatidae. **Text S2.** Description of ecological niche for the Belostomatidae across Africa. **Figure S9.** Environmental suitability for the Notonectidae across Africa and prediction uncertainty (95% CI). **Figure S10.** Predicted occurrence for the Notonectidae across Africa and uncertainty. **Figure S11.** Partial dependence plots of the relative contribution of covariates to the boosted regression tree (BRT) model for the Notonectidae. **Figure S12.** Partial dependence plots of the relative contribution of covariates to the random forest (RF) model for the Notonectidae. **Text S3.** Description of ecological niche for the Notonectidae across Africa. **Figure S13.** Environmental suitability for the Nepidae across Africa and prediction uncertainty (95% CI). **Figure S14.** Predicted occurrence for the Nepidae across Africa and uncertainty. **Figure S15.** Partial dependence plots of the relative contribution of covariates to the boosted regression tree (BRT) model for the Nepidae. **Figure S16.** Partial dependence plots of the relative contribution of covariates to the random forest (RF) model for the Nepidae. **Text S4.** Description of ecological niche for the Nepidae across Africa. **Figure S17.** Environmental suitability for the Corixidae across Africa and prediction uncertainty (95% CI). **Figure S18.** Predicted occurrence for the Corixidae across Africa and uncertainty. **Figure S19.** Partial dependence plots of the relative contribution of covariates to the boosted regression tree (BRT) model for the Corixidae. **Figure S20.** Partial dependence plots of the relative contribution of covariates to the random forest (RF) model for the Corixidae. **Text S5.** Description of ecological niche for the Corixidae across Africa. **Figure S21.** Environmental suitability for the Gerridae across Africa and prediction uncertainty (95% CI). **Figure S22.** Predicted occurrence for the Gerridae across Africa and uncertainty. **Figure S23.** Partial dependence plots of the relative contribution of covariates to the boosted regression tree (BRT) model for the Gerridae. **Figure S24.** Partial dependence plots of the relative contribution of covariates to the random forest (RF) model for the Gerridae. **Text S6.** Description of ecological niche for the Gerridae across Africa. (PDF 4223 kb)

## Abbreviations

ANN: Artificial Neural Networks
AUC: Area under the curve of the receiver operation characteristic (ROC)
BU: Buruli ulcer
CGIAR-CSI: Consortium for Spatial Information
DEM: Digital Elevation Model
ENFA: Ecological Niche Factor analysis
EVI: Enhanced Vegetation Index
GAM: Generalized Additive Models
GBIF: Global Biodiversity Information Facility database (GBIF)
GBM: Generalized Boosted regression trees Models
MARS: Multiple Adaptive Regression Splines
MaxEnt: Maximum Entropy models
NDVI: Normalized Difference Vegetation Index
PCC: Positive Correctly Classified
PET: Potential Evapo-transpiration
RF: Random Forest
SRTM: Shuttle Radar Topographic Mission
TCW: Topographic Wetness Index
TSS: True Skill Statistic
VIF: Variance Inflation Factor
WHO: World Health Organization
