# Supplementary material for: Modelling the spatial distribution of aquatic insects (Order Hemiptera) potentially involved in the transmission of Mycobacterium ulcerans in Africa
Source: Parasit Vectors. 2018 Sep 6;11:501. doi: 10.1186/s13071-018-3066-3 (PMC6127916; doi:10.1186/s13071-018-3066-3)

**Additional file 1**

**Table S1.** **Median and 95% confidence intervals of validation indicators (TSS, AUC and PCC) for the modelling approaches tested: generalized linear models (GLM), generalized additive models (GAM), boosted regression trees (GBM), artificial neural networks (ANN), multiple adaptive regression splines (MARS), maximum entropy (MAXENT) and random forest (RF).** TSS: true skill statistic, ROC: the area under the receiver operation characteristic (ROC) curve, and PCC: positive (“occurrence”) correctly classified.

**Figure S1. Distribution of communities reporting Buruli ulcer in Ghana (2007-2010) and Cameroon (2003-2015).** Communities are displayed over a gridded map of predicted distribution of Hemiptera families.

**
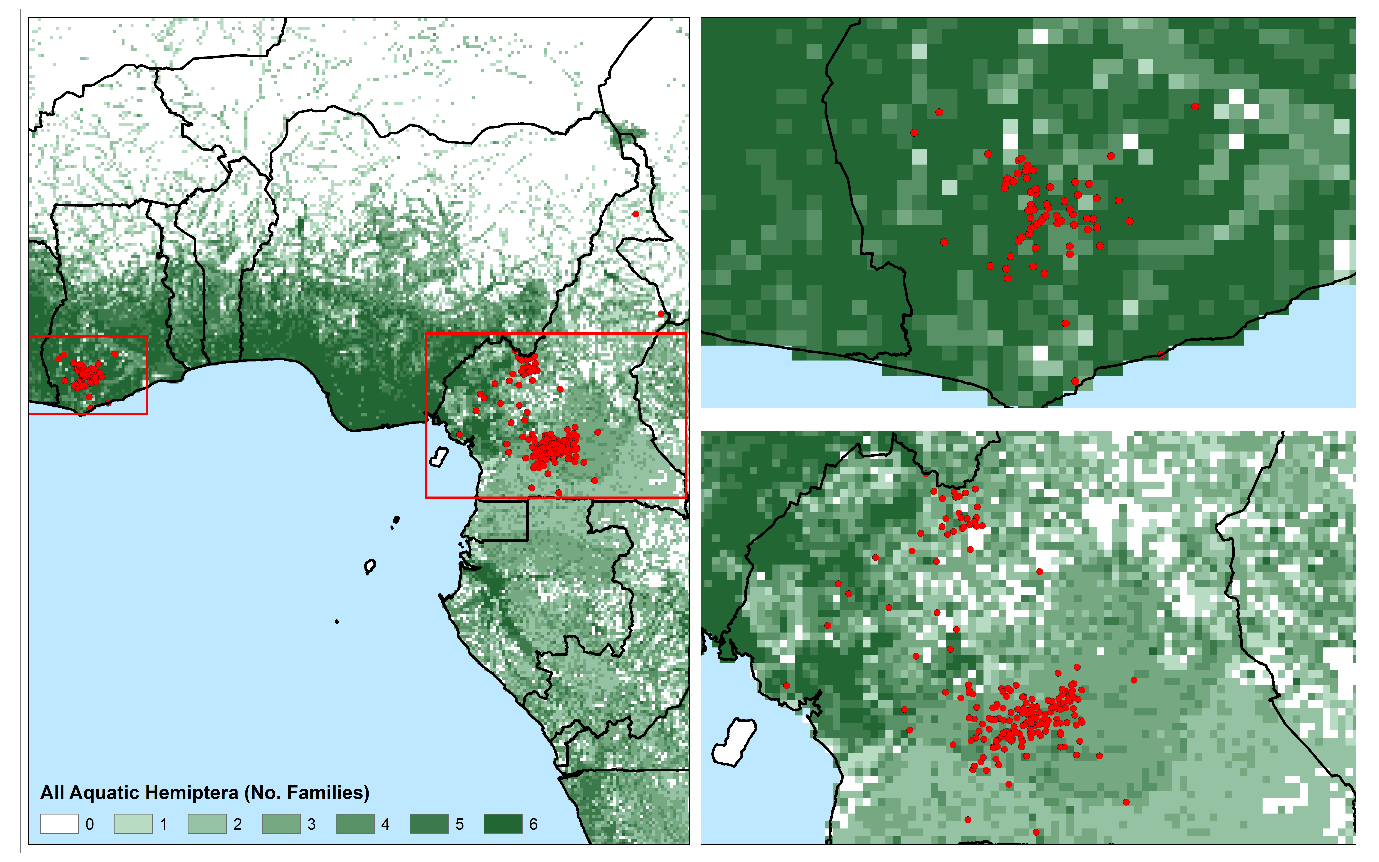
**

**Figure S2. Variable contribution of final ensemble models based on *boosted regression trees*  and *random forest* for Fam. Naucoridae (A), Fam. Belostomatidae (B) and Fam. Notonectidae (C).** Variable contribution is provided as percentage, and it shows the relative contribution of selected environmental predictors to the final model.

**
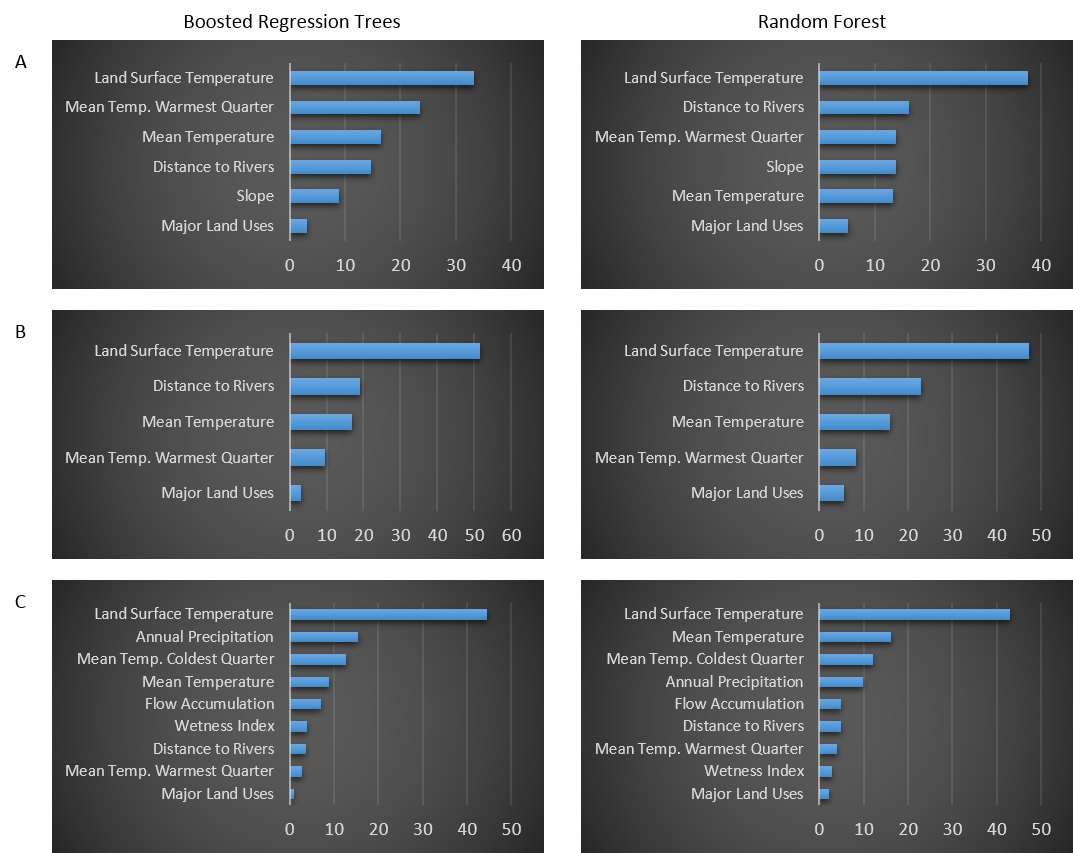
**

**Figure S3. Variable contribution of final ensemble models based on *boosted regression trees*  and *random forest* for Fam. Nepidae (A), Fam. Corixidae (B) and Fam. Gerridae (C).** Variable contribution is provided as percentage, and it shows the relative contribution of selected environmental predictors to the final ensemble model.


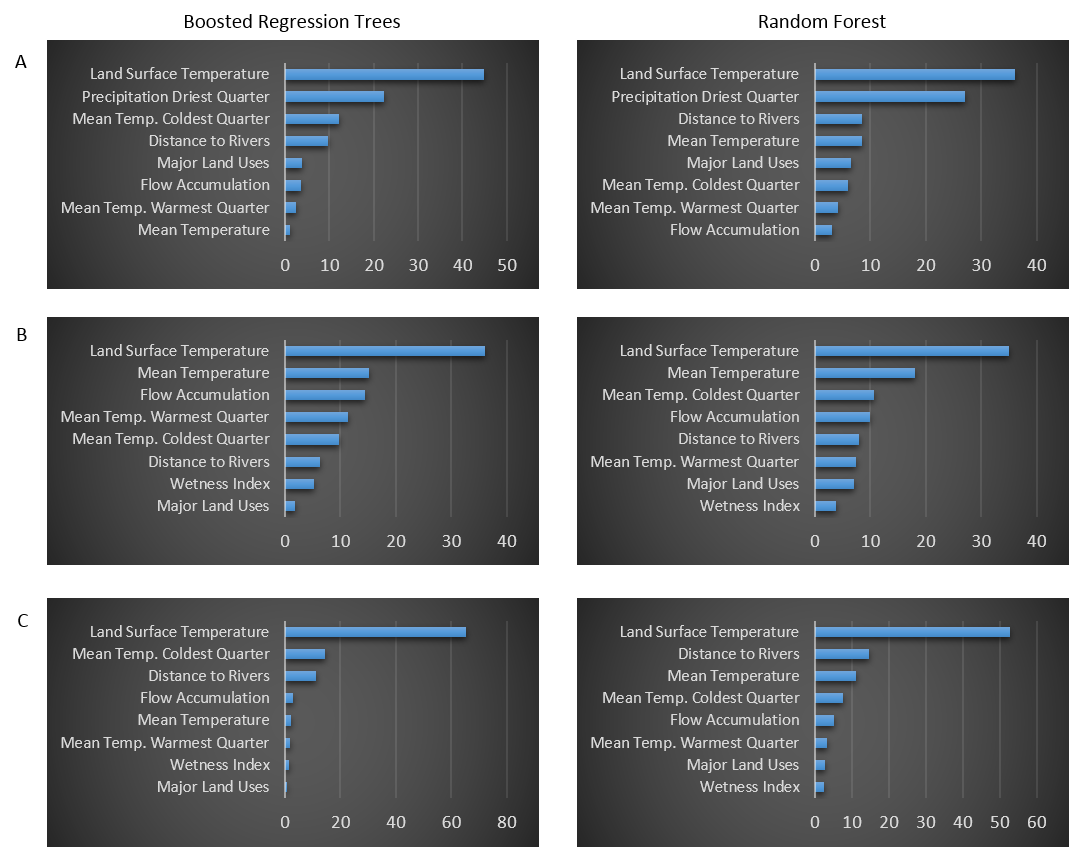

Supplement: Supplementary file 1 — Table S1. Median and 95% confidence intervals of validation indicators (TSS, AUC and PCC) for the modelling approaches tested: generalized linear models (GLM), generalized additive models (GAM), boosted regression trees (GBM), artificial neural networks (ANN), multiple adaptive regression splines (MARS), maximum entropy (MAXENT) and random forest (RF). Figure S1. Distribution of communities reporting Buruli ulcer in Ghana (2007–2010) and Cameroon (2003–2015) over a gridded map of predicted distribution of Hemiptera families. Figure S2. Variable contribution of final ensemble models based on GBM and RF algorithms for the Naucoridae, Belostomatidae and Notonectidae. Figure S3. Variable contribution of final ensemble models based on GBM and RF algorithms for the Nepidae, Corixidae and Gerridae. (DOCX 795 kb) [file 13071_2018_3066_MOESM1_ESM.docx]
