# Supplementary material for: Modelling the spatial distribution of aquatic insects (Order Hemiptera) potentially involved in the transmission of Mycobacterium ulcerans in Africa
Source: Parasit Vectors. 2018 Sep 6;11:501. doi: 10.1186/s13071-018-3066-3 (PMC6127916; doi:10.1186/s13071-018-3066-3)
Supplement: Supplementary file 3 — Figure S1. Environmental suitability for the Naucoridae across Africa and prediction uncertainty (95% CI). Figure S2. Predicted occurrence for the Naucoridae across Africa and uncertainty. Figure S3. Partial dependence plots of the relative contribution of covariates to the boosted regression tree (BRT) model for the Naucoridae. Figure S4. Partial dependence plots of the relative contribution of covariates to the random forest (RF) model for the Naucoridae. Text S1. Description of ecological niche for the Naucoridae across Africa. Figure S5. Environmental suitability for the Belostomatidae across Africa and prediction uncertainty (95% CI). Figure S6. Predicted occurrence for the Belostomatidae across Africa and uncertainty. Figure S7. Partial dependence plots of the relative contribution of covariates to the boosted regression tree (BRT) model for the Belostomatidae. Figure S8. Partial dependence plots of the relative contribution of covariates to the random forest (RF) model for the Belostomatidae. Text S2. Description of ecological niche for the Belostomatidae across Africa. Figure S9. Environmental suitability for the Notonectidae across Africa and prediction uncertainty (95% CI). Figure S10. Predicted occurrence for the Notonectidae across Africa and uncertainty. Figure S11. Partial dependence plots of the relative contribution of covariates to the boosted regression tree (BRT) model for the Notonectidae. Figure S12. Partial dependence plots of the relative contribution of covariates to the random forest (RF) model for the Notonectidae. Text S3. Description of ecological niche for the Notonectidae across Africa. Figure S13. Environmental suitability for the Nepidae across Africa and prediction uncertainty (95% CI). Figure S14. Predicted occurrence for the Nepidae across Africa and uncertainty. Figure S15. Partial dependence plots of the relative contribution of covariates to the boosted regression tree (BRT) model for the Nepidae. Figure S16. Partial dependence [file 13071_2018_3066_MOESM3_ESM.pdf]

Modelling the spatial distribution of aquatic insects (Order Hemiptera) potentially involved in the transmission of *Mycobacterium ulcerans* in Africa

Jorge Cano, Antonio Rodriguez, Hope Simpson, Earnest Njih, Jose F. Gómez & Rachel L Pullan

Additional file 3. Modelling outcomes for every Hemiptera family included in this study: Naucoridae, Belostomatidae, Notonectidae, Nepidae, Corixidae and Gerridae.

Naucoridae

Figure S1. Environmental suitability for Fam. Naucoridae across Africa and prediction uncertainty (95% confidence interval). *Insect image from Wikimedia Commons*

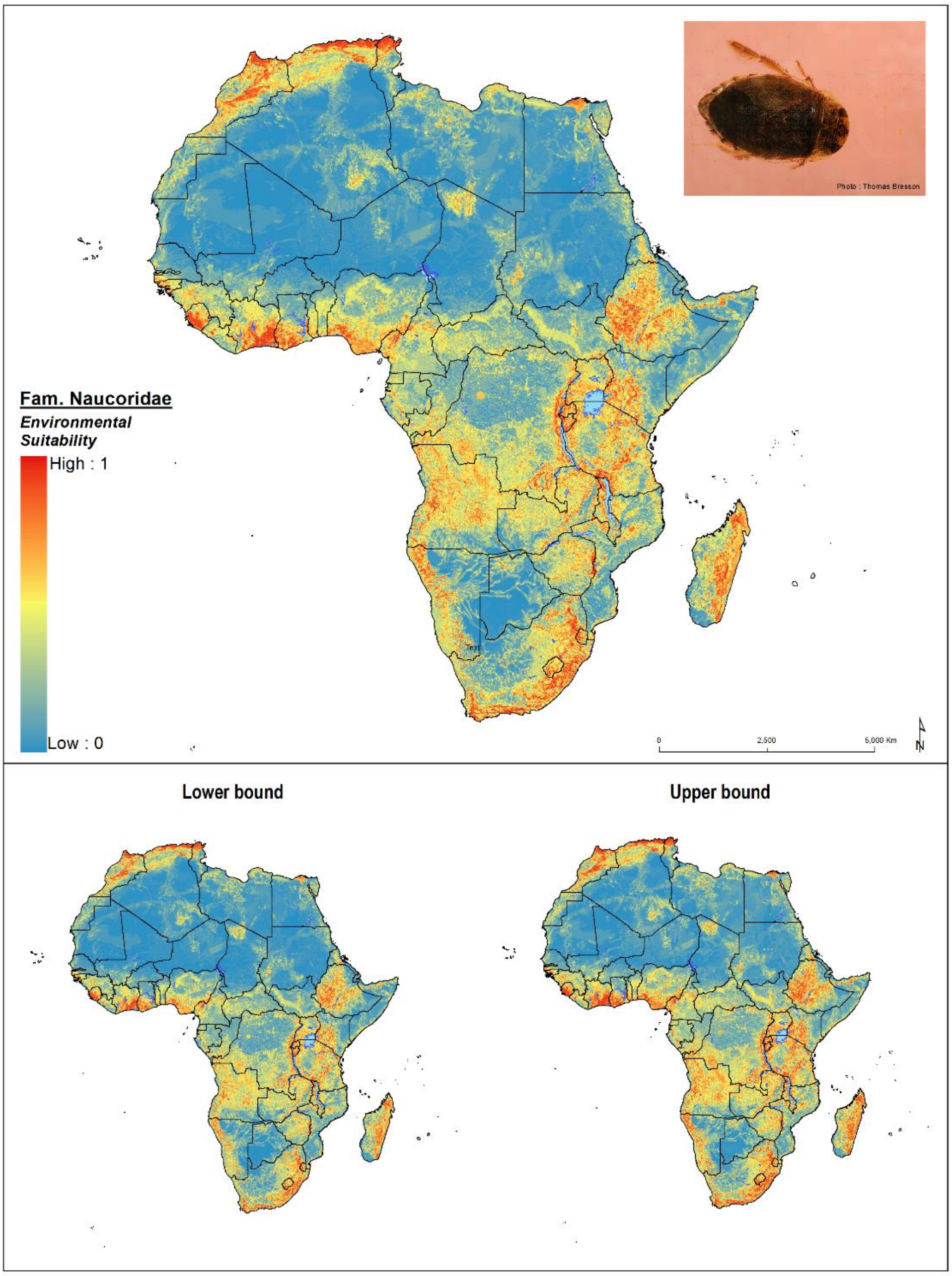

Modelling the spatial distribution of aquatic insects (Order Hemiptera) potentially involved in the transmission of *Mycobacterium ulcerans* in Africa

Jorge Cano, Antonio Rodriguez, Hope Simpson, Earnest Njih, Jose F. Gómez & Rachel L Pullan

Figure S2. Predicted occurrence for Fam. Naucoridae across Africa and uncertainty. Optimal threshold was fitted to get better trade-off between sensitivity, specificity and proportion correctly classified (PCC). *Insect image from Wikimedia Commons*

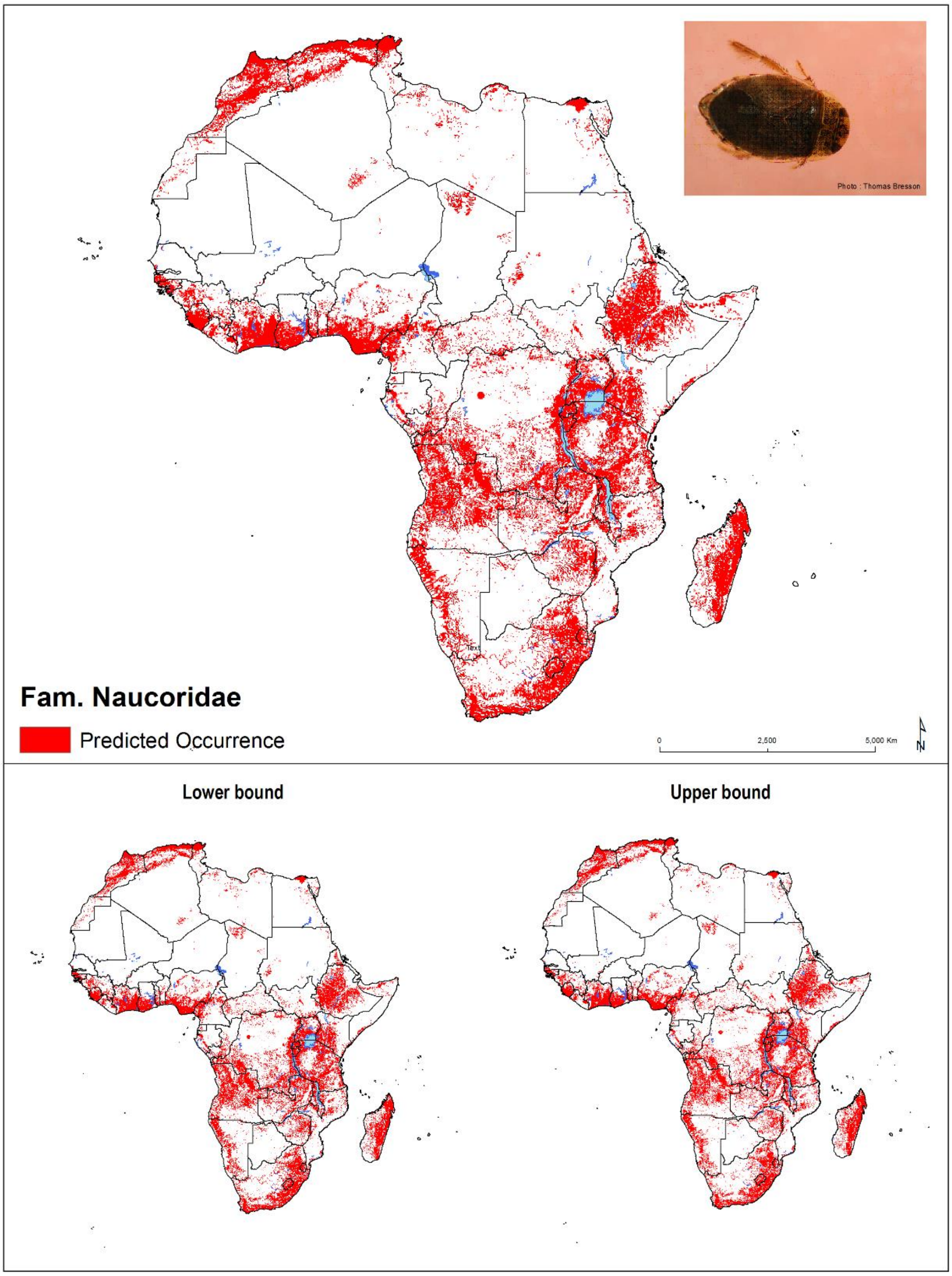

Modelling the spatial distribution of aquatic insects (Order Hemiptera) potentially involved in the transmission of *Mycobacterium ulcerans* in Africa

Jorge Cano, Antonio Rodriguez, Hope Simpson, Earnest Njih, Jose F. Gómez & Rachel L Pullan

Figure S3. Partial dependence plots of the relative contribution of covariates to the boosted regression tree (BRT) model for Fam. Naucoridae, averaged over 80 ensembles. Blue lines represent the mean partial dependence over all 80 BRT ensembles and grey envelopes the standard deviation from the mean. The *y*-axis is the transformed logit response and *x*-axis is the full range of covariates values.

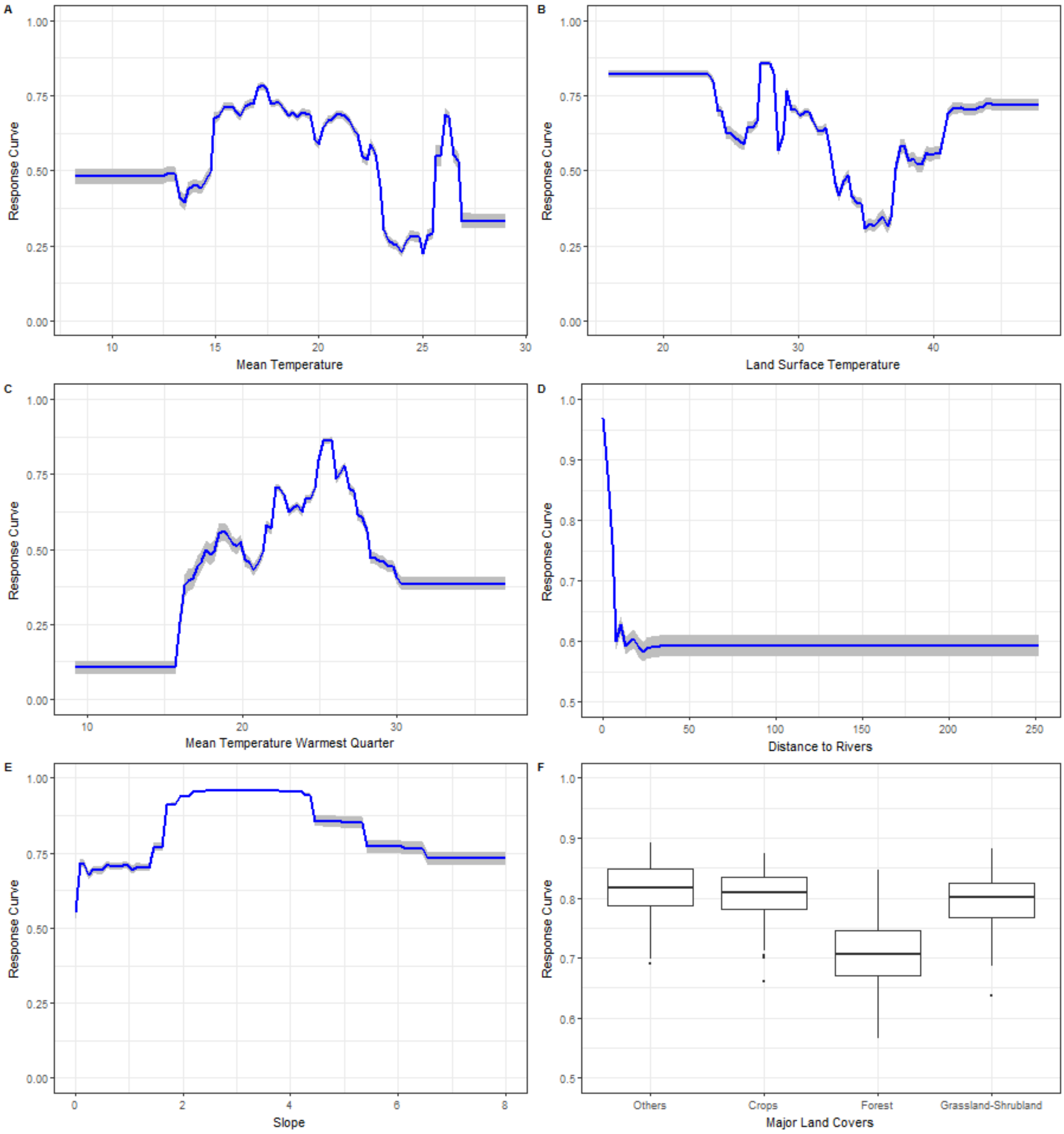

Modelling the spatial distribution of aquatic insects (Order Hemiptera) potentially involved in the transmission of *Mycobacterium ulcerans* in Africa

Jorge Cano, Antonio Rodriguez, Hope Simpson, Earnest Njih, Jose F. Gómez & Rachel L Pullan

Figure S4. Partial dependence plots of the relative contribution of covariates to the random forest (RF) model for Fam. Naucoridae, averaged over 80 ensembles. Blue lines represent the mean partial dependence over all 80 RF ensembles and grey envelopes the standard deviation from the mean. The *y*-axis is the transformed logit response and *x*-axis is the full range of covariates values.

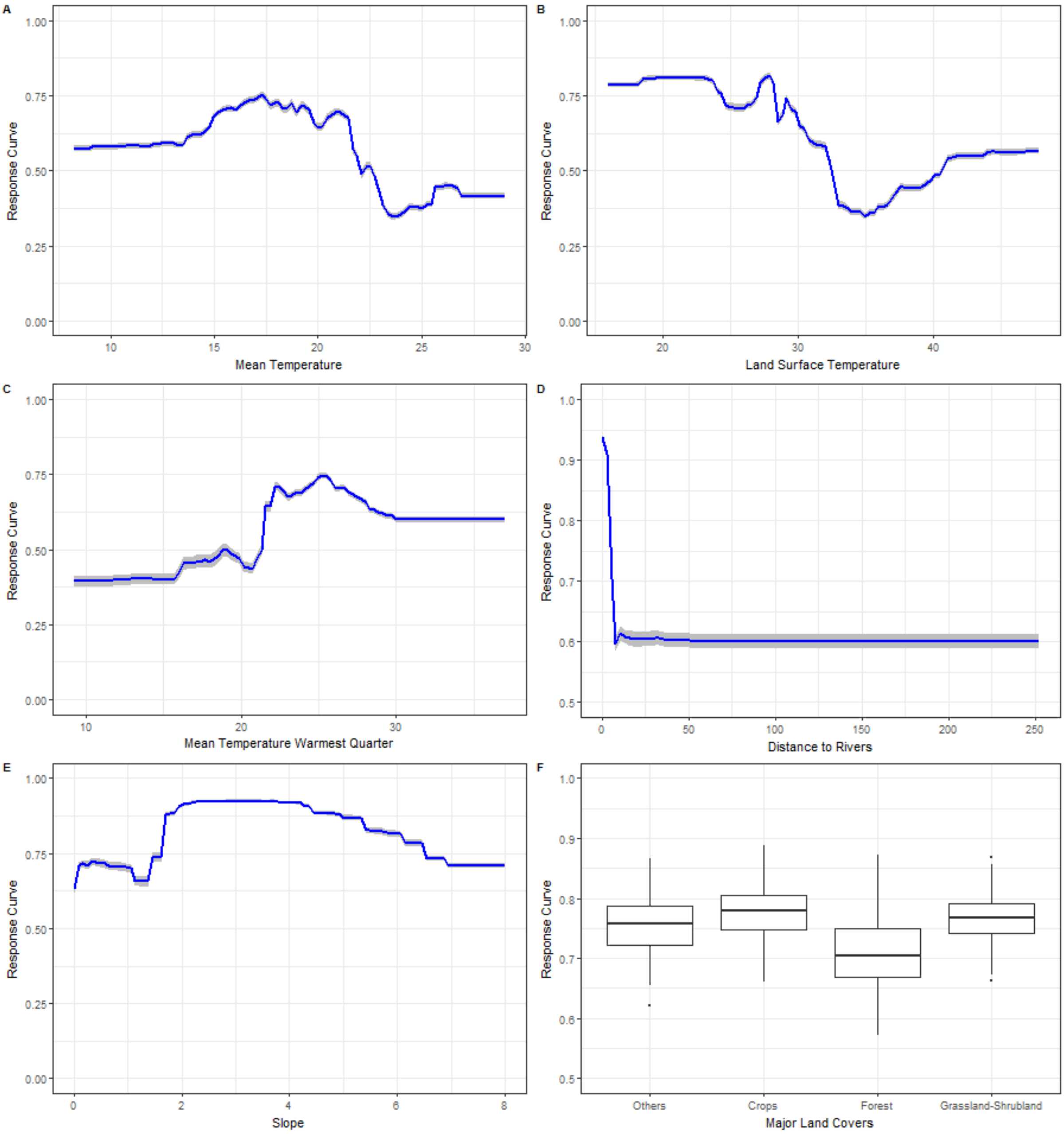

Modelling the spatial distribution of aquatic insects (Order Hemiptera) potentially involved in the transmission of *Mycobacterium ulcerans* in Africa

Jorge Cano, Antonio Rodriguez, Hope Simpson, Earnest Njih, Jose F. Gómez & Rachel L Pullan

Text S1. Description of ecological niche for the Hemiptera insects of the Fam. Naucoridae across Africa.

Species of this family of aquatic Hemiptera show to be widely present across Africa, except throughout drier and hotter areas such as Kalahari and Sahara deserts. Our model predicts their presence in coastal areas of West Africa and large areas of Eastern Africa (i.e. Uganda, Ethiopia, northeastern Kenya and Tanzania). Upper Congo basin seems not to be a suitable ecological niche for this water bug family and western coast of Madagascar.

The marginal effect plots show a preference by transformed environments such as agriculture landscape over forest and grassland areas, and its presence is strongly link to streams. Its optimal temperature goes from 15°C to 20°C degrees, and at land surface temperatures above 28°C the suitability of the environment declines sharply.

Modelling the spatial distribution of aquatic insects (Order Hemiptera) potentially involved in the transmission of *Mycobacterium ulcerans* in Africa

Jorge Cano, Antonio Rodriguez, Hope Simpson, Earnest Njih, Jose F. Gómez & Rachel L Pullan

Belostomatidae

Figure S5. Environmental suitability for Fam. Belostomatidae across Africa and prediction uncertainty (95% confidence interval). *Insect image from Wikimedia Commons*

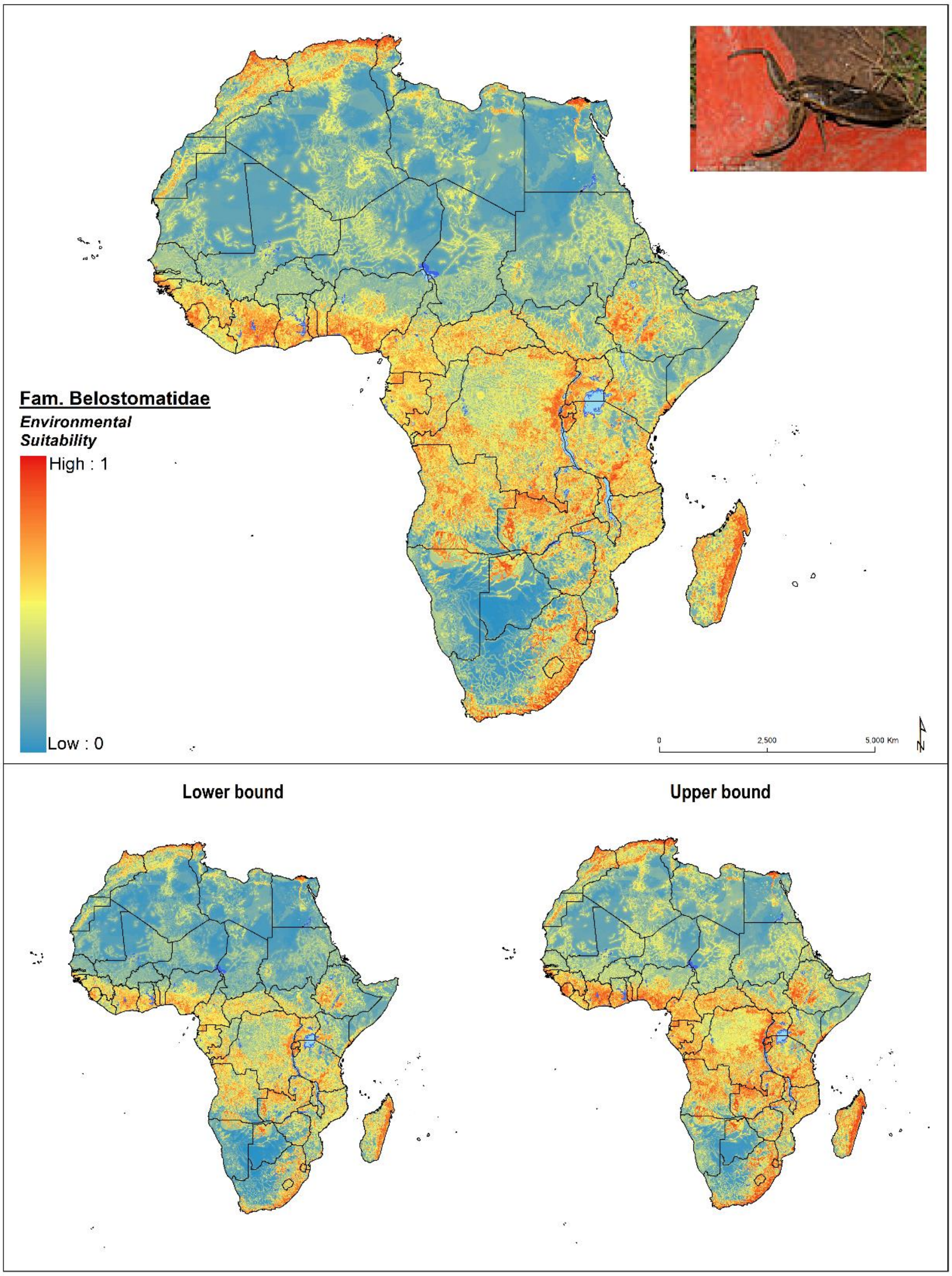

Modelling the spatial distribution of aquatic insects (Order Hemiptera) potentially involved in the transmission of *Mycobacterium ulcerans* in Africa

Jorge Cano, Antonio Rodriguez, Hope Simpson, Earnest Njih, Jose F. Gómez & Rachel L Pullan

Figure S6. Predicted occurrence for Fam. Belostomatidae across Africa and uncertainty. Optimal threshold was fitted to get better trade-off between sensitivity, specificity and proportion correctly classified (PCC). *Insect image from Wikimedia Commons*

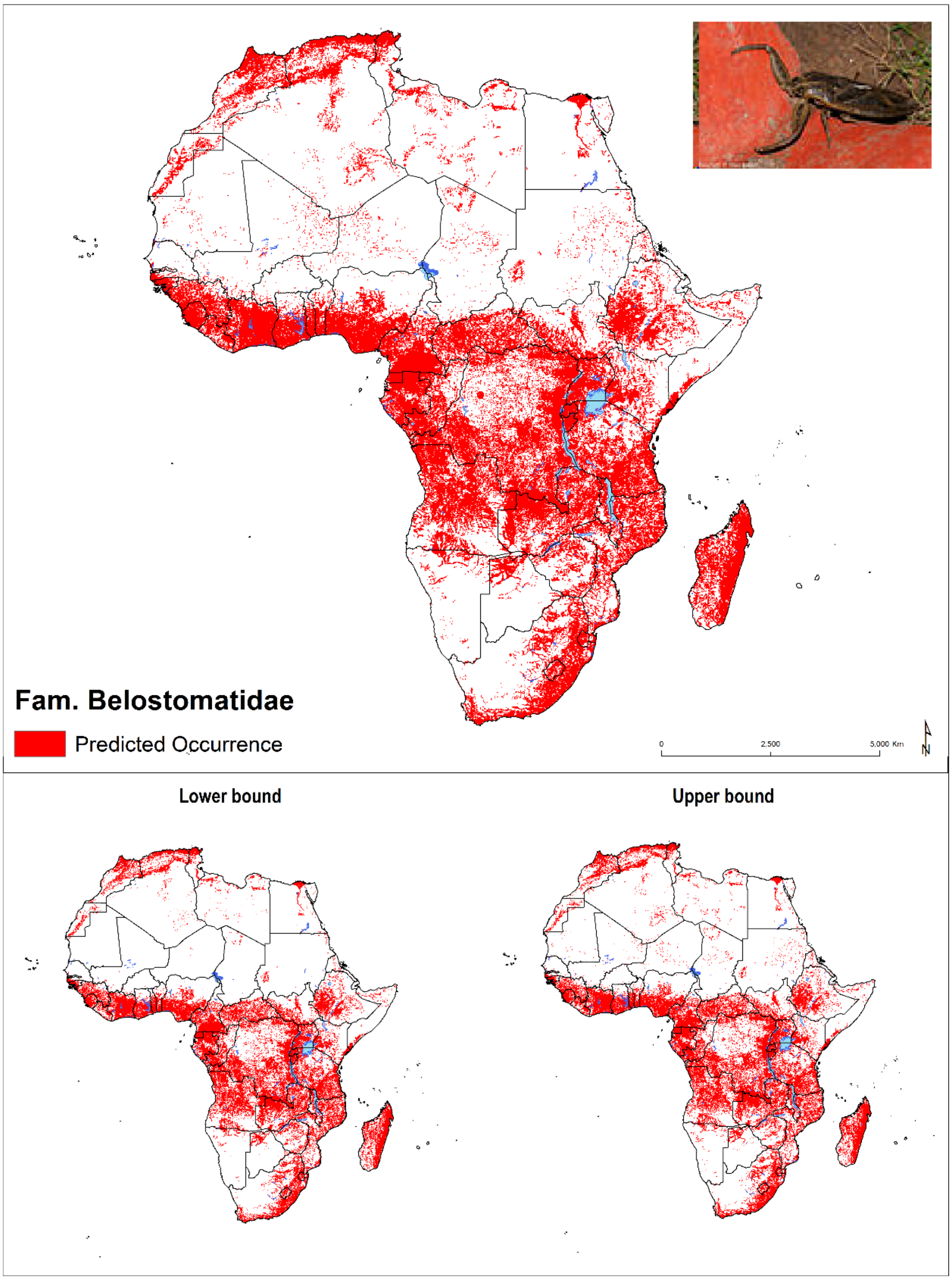

Modelling the spatial distribution of aquatic insects (Order Hemiptera) potentially involved in the transmission of *Mycobacterium ulcerans* in Africa

Jorge Cano, Antonio Rodriguez, Hope Simpson, Earnest Njih, Jose F. Gómez & Rachel L Pullan

Figure S7. Partial dependence plots of the relative contribution of covariates to the boosted regression tree (BRT) model for Fam. Belostomatidae, averaged over 80 ensembles. Blue lines represent the mean partial dependence over all 80 BRT ensembles and grey envelopes the standard deviation from the mean. The *y*-axis is the transformed logit response and *x*-axis is the full range of covariates values.

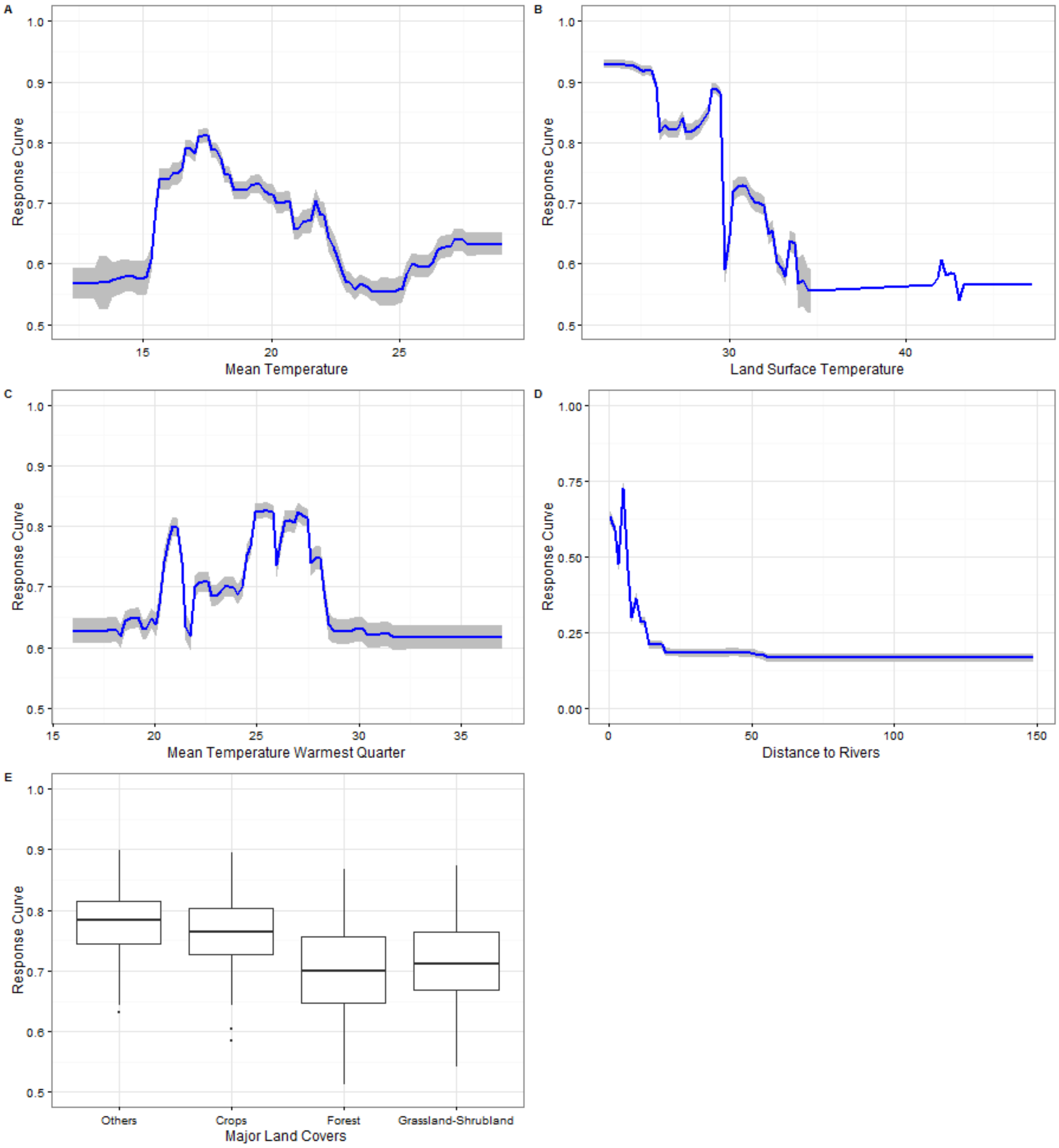

Modelling the spatial distribution of aquatic insects (Order Hemiptera) potentially involved in the transmission of *Mycobacterium ulcerans* in Africa

Jorge Cano, Antonio Rodriguez, Hope Simpson, Earnest Njih, Jose F. Gómez & Rachel L Pullan

Figure S8. Partial dependence plots of the relative contribution of covariates to the random forest (RF) model for Fam. Belostomatidae, averaged over 80 ensembles. Blue lines represent the mean partial dependence over all 80 RF ensembles and grey envelopes the standard deviation from the mean. The *y*-axis is the transformed logit response and *x*-axis is the full range of covariates values.

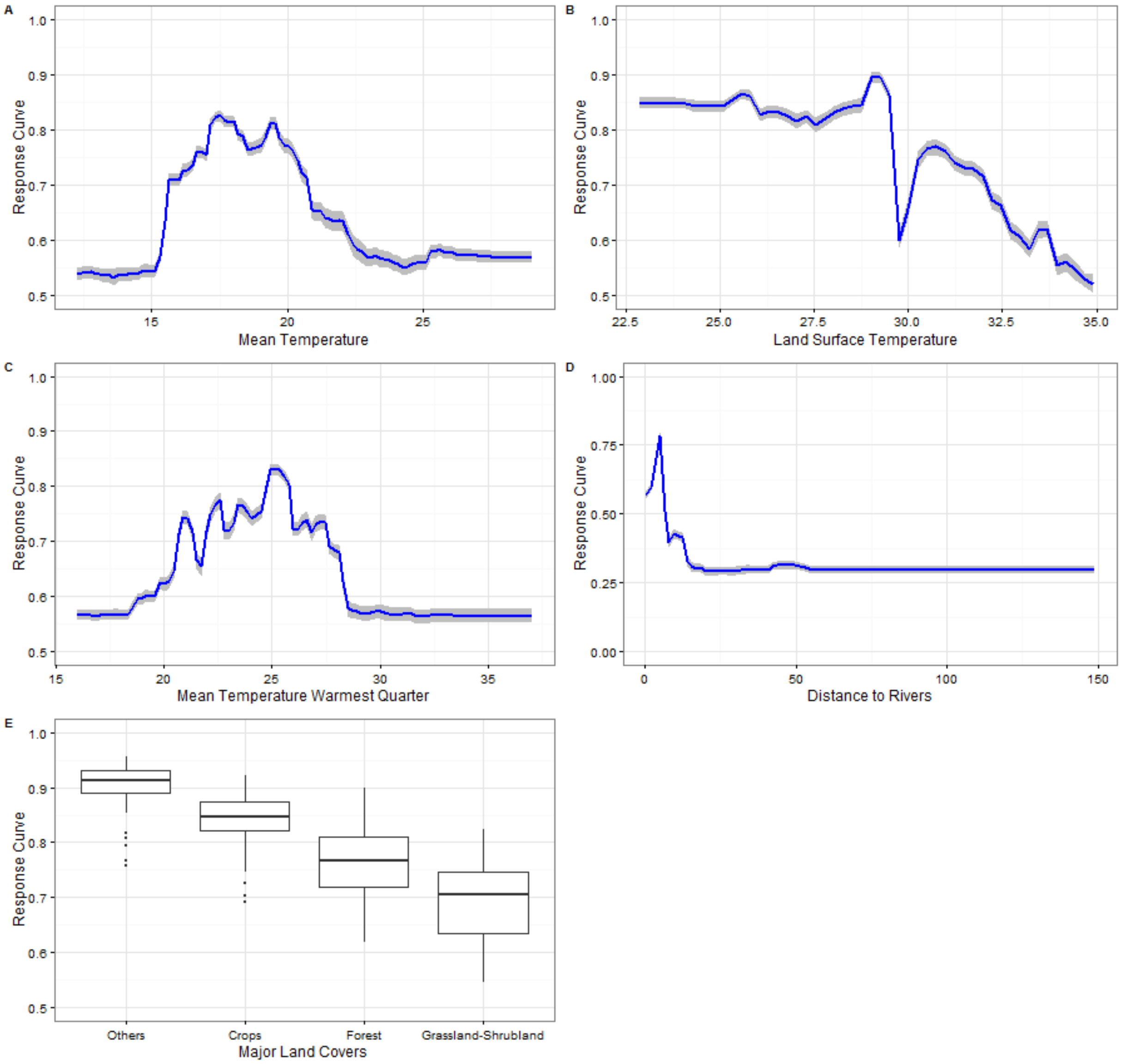

Modelling the spatial distribution of aquatic insects (Order Hemiptera) potentially involved in the transmission of *Mycobacterium ulcerans* in Africa

Jorge Cano, Antonio Rodriguez, Hope Simpson, Earnest Njih, Jose F. Gómez & Rachel L Pullan

Text S2. Description of ecological niche for the Hemiptera insects of the Fam. Belostomatidae across Africa.

According to our model, this Hemiptera family shows a ubiquitous geographical distribution across Africa. It is widely distributed in western Africa, where appears to occupy not only coastal areas but also inland areas. Drier areas limiting with the Sahara desert from Cameroon, Nigeria, Central African Republic (CAR) and the nearly whole Namibia seems to be unsuitable environments for species of this family. Also according to the ensemble model, it would be present in all middle Africa.

As we observed in Naucoridae family, its distribution appears to be strongly mediated by temperature, showing preference for mean temperatures between 16°C and 23°C degrees and close to streams. Belostomatidae species seems to prefer human-transformed landscapes ("other" land cover category comprises urban and bare areas) followed by crops, what it can favour the contact with population.

Modelling the spatial distribution of aquatic insects (Order Hemiptera) potentially involved in the transmission of *Mycobacterium ulcerans* in Africa

Jorge Cano, Antonio Rodriguez, Hope Simpson, Earnest Njih, Jose F. Gómez & Rachel L Pullan

Notonectidae

Figure S9. Environmental suitability for Fam. Notonectidae across Africa and prediction uncertainty (95% confidence interval). *Insect image from Wikimedia Commons*

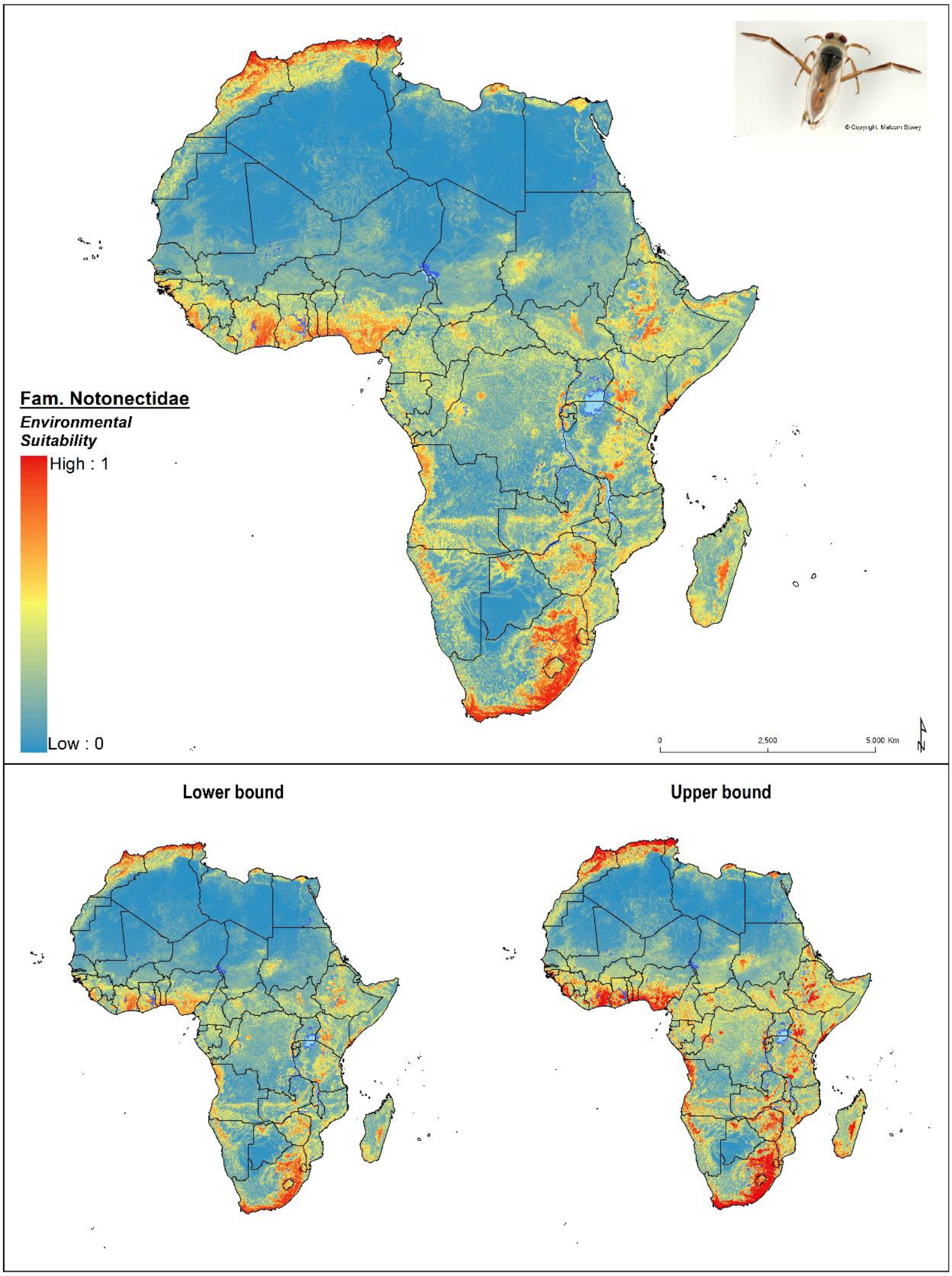

Modelling the spatial distribution of aquatic insects (Order Hemiptera) potentially involved in the transmission of *Mycobacterium ulcerans* in Africa

Jorge Cano, Antonio Rodriguez, Hope Simpson, Earnest Njih, Jose F. Gómez & Rachel L Pullan

Figure S10. Predicted occurrence for Fam. Notonectidae across Africa and uncertainty. Optimal threshold was fitted to get better trade-off between sensitivity, specificity and proportion correctly classified (PCC). *Insect image from Wikimedia Commons*

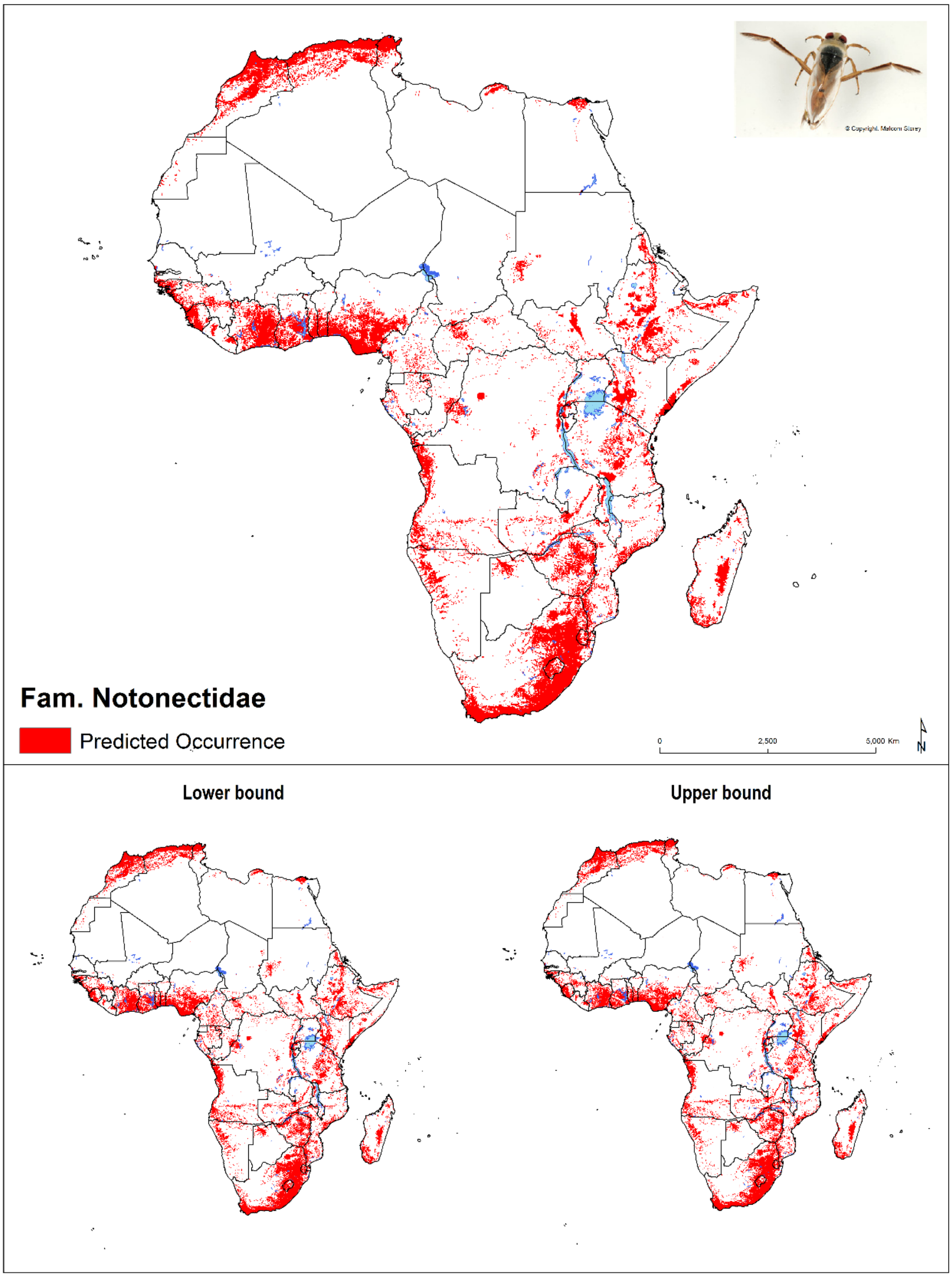

Modelling the spatial distribution of aquatic insects (Order Hemiptera) potentially involved in the transmission of *Mycobacterium ulcerans* in Africa

Jorge Cano, Antonio Rodriguez, Hope Simpson, Earnest Njih, Jose F. Gómez & Rachel L Pullan

Figure S11. Partial dependence plots of the relative contribution of covariates to the boosted regression tree (BRT) model for Fam. Notonectidae, averaged over 80 ensembles. Blue lines represent the mean partial dependence over all 80 BRT ensembles and grey envelopes the standard deviation from the mean. The *y*-axis is the transformed logit response and *x*-axis is the full range of covariates values.

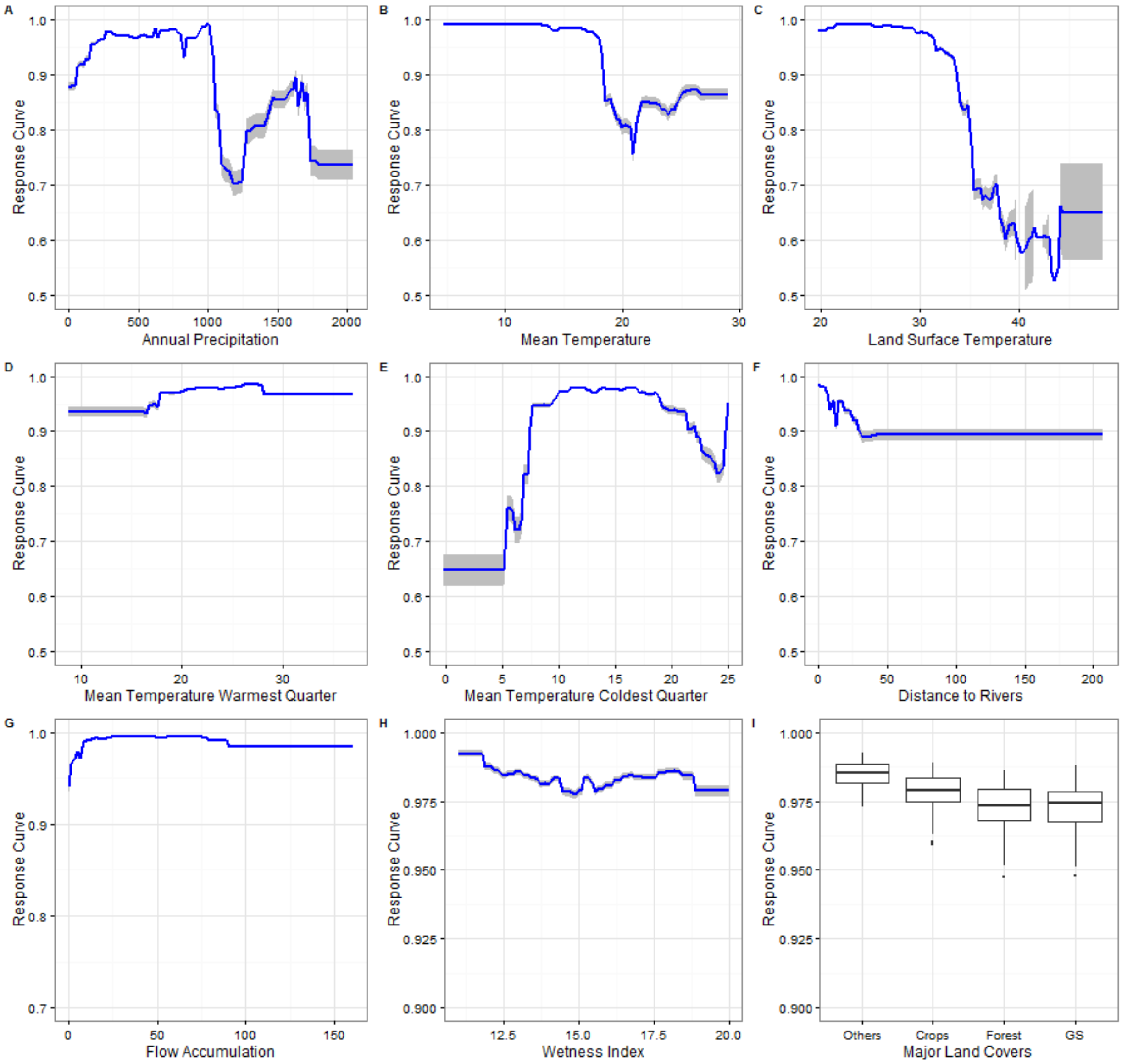

Modelling the spatial distribution of aquatic insects (Order Hemiptera) potentially involved in the transmission of *Mycobacterium ulcerans* in Africa

Jorge Cano, Antonio Rodriguez, Hope Simpson, Earnest Njih, Jose F. Gómez & Rachel L Pullan

Figure S12. Partial dependence plots of the relative contribution of covariates to the random forest (RF) model for Fam. Notonectidae, averaged over 80 ensembles. Blue lines represent the mean partial dependence over all 80 RF ensembles and grey envelopes the standard deviation from the mean. The *y*-axis is the transformed logit response and *x*-axis is the full range of covariates values.

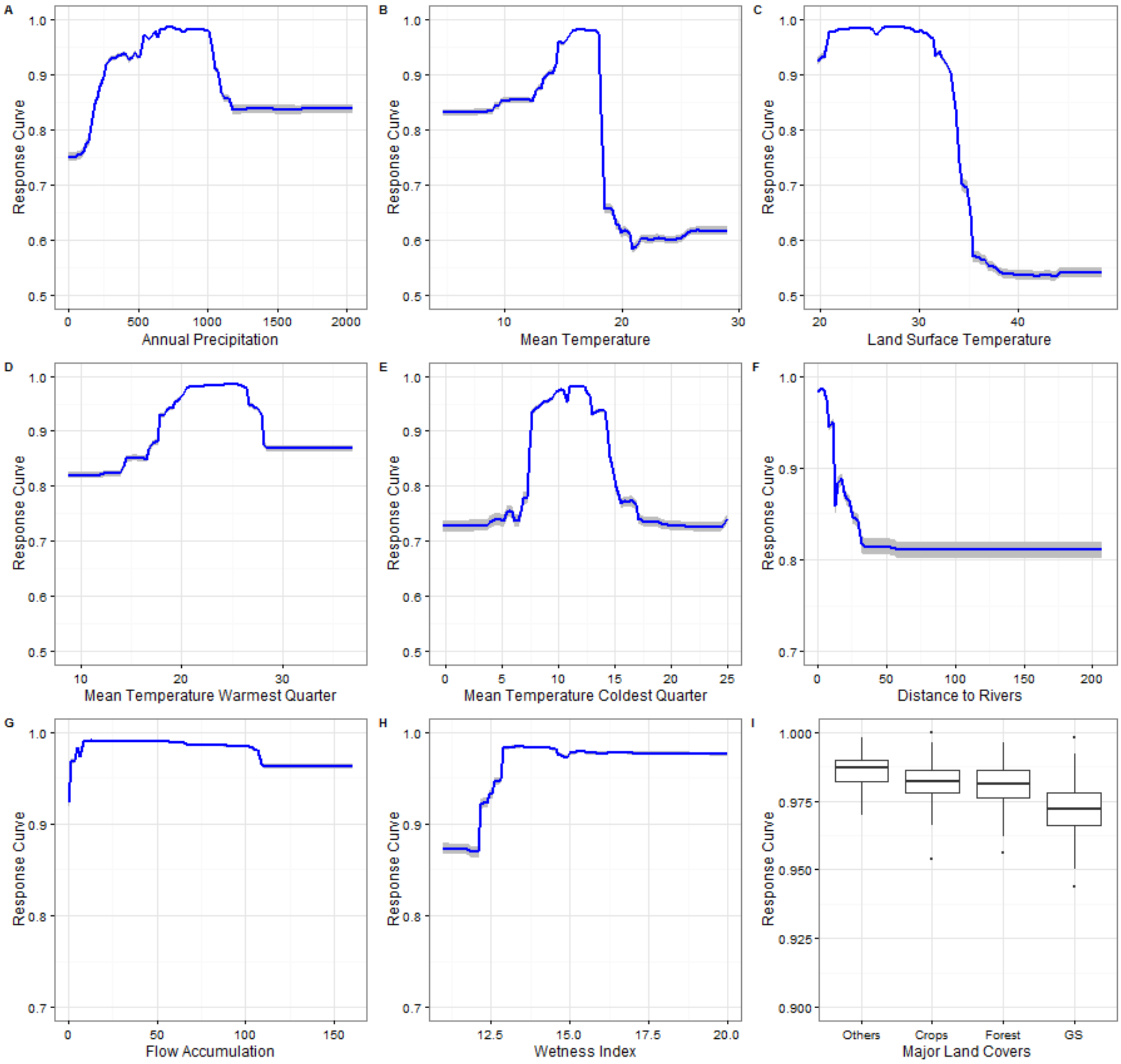

Modelling the spatial distribution of aquatic insects (Order Hemiptera) potentially involved in the transmission of *Mycobacterium ulcerans* in Africa

Jorge Cano, Antonio Rodriguez, Hope Simpson, Earnest Njih, Jose F. Gómez & Rachel L Pullan

Text S3. Description of ecological niche for the Hemiptera insects of the Fam. Notonectidae across Africa.

Notonectidae species seems to have a more restricted distribution in Africa, being more predominant in western and southern Africa but narrowly distributed in middle Africa. In eastern Africa (Ethiopia, Kenya, Tanzania and Madagascar), it seems to be present at high altitude areas, whereas it would be distributing along coastal areas of western and southern Africa (i.e. Angola).

The marginal effect plots show this Hemiptera family to be more resistant to higher LST, even though environment suitability sharply declines from 35°C. Surprisingly, optimal environmental mean temperature for these water bugs varies between 10°C and 20°C degrees, what likely is making its distribution more restricted comparing to Naucoridae and Notonectidae species. Environmental suitability for this family is also driven by moderate annual precipitation (350 – 1,000 mm/annual) and they seem to inhabit all type of land covers with major preference for human-made landscapes (i.e. urban settings and agricultural lands).

Modelling the spatial distribution of aquatic insects (Order Hemiptera) potentially involved in the transmission of *Mycobacterium ulcerans* in Africa

Jorge Cano, Antonio Rodriguez, Hope Simpson, Earnest Njih, Jose F. Gómez & Rachel L Pullan

Nepidae

Figure S13. Environmental suitability for Fam. Nepidae across Africa and prediction uncertainty (95% confidence interval). *Insect image from Wikimedia Commons*

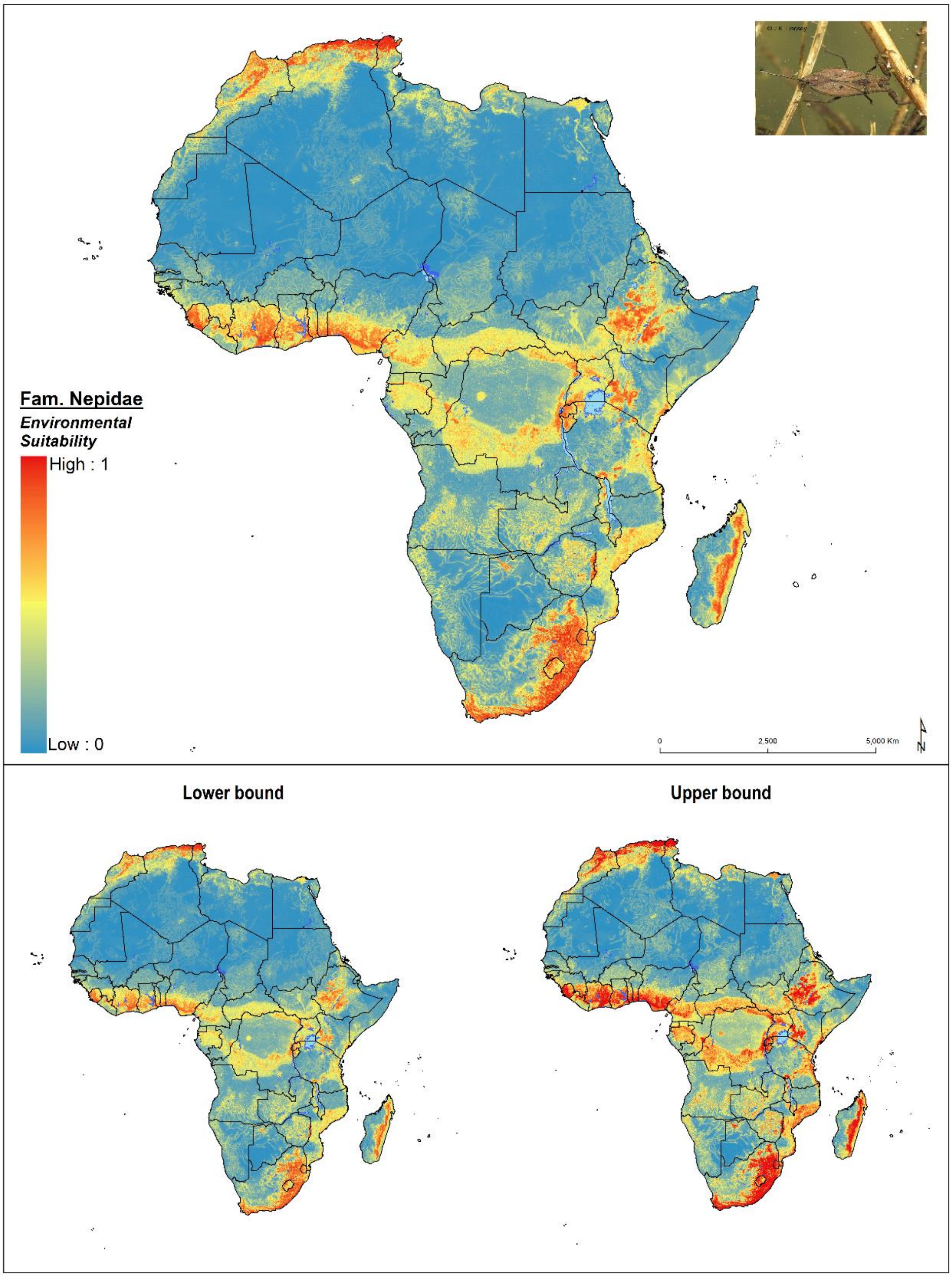

Modelling the spatial distribution of aquatic insects (Order Hemiptera) potentially involved in the transmission of *Mycobacterium ulcerans* in Africa

Jorge Cano, Antonio Rodriguez, Hope Simpson, Earnest Njih, Jose F. Gómez & Rachel L Pullan

Figure S14. Predicted occurrence for Fam. Nepidae across Africa and uncertainty. Optimal threshold was fitted to get better trade-off between sensitivity, specificity and proportion correctly classified (PCC). *Insect image from Wikimedia Commons*

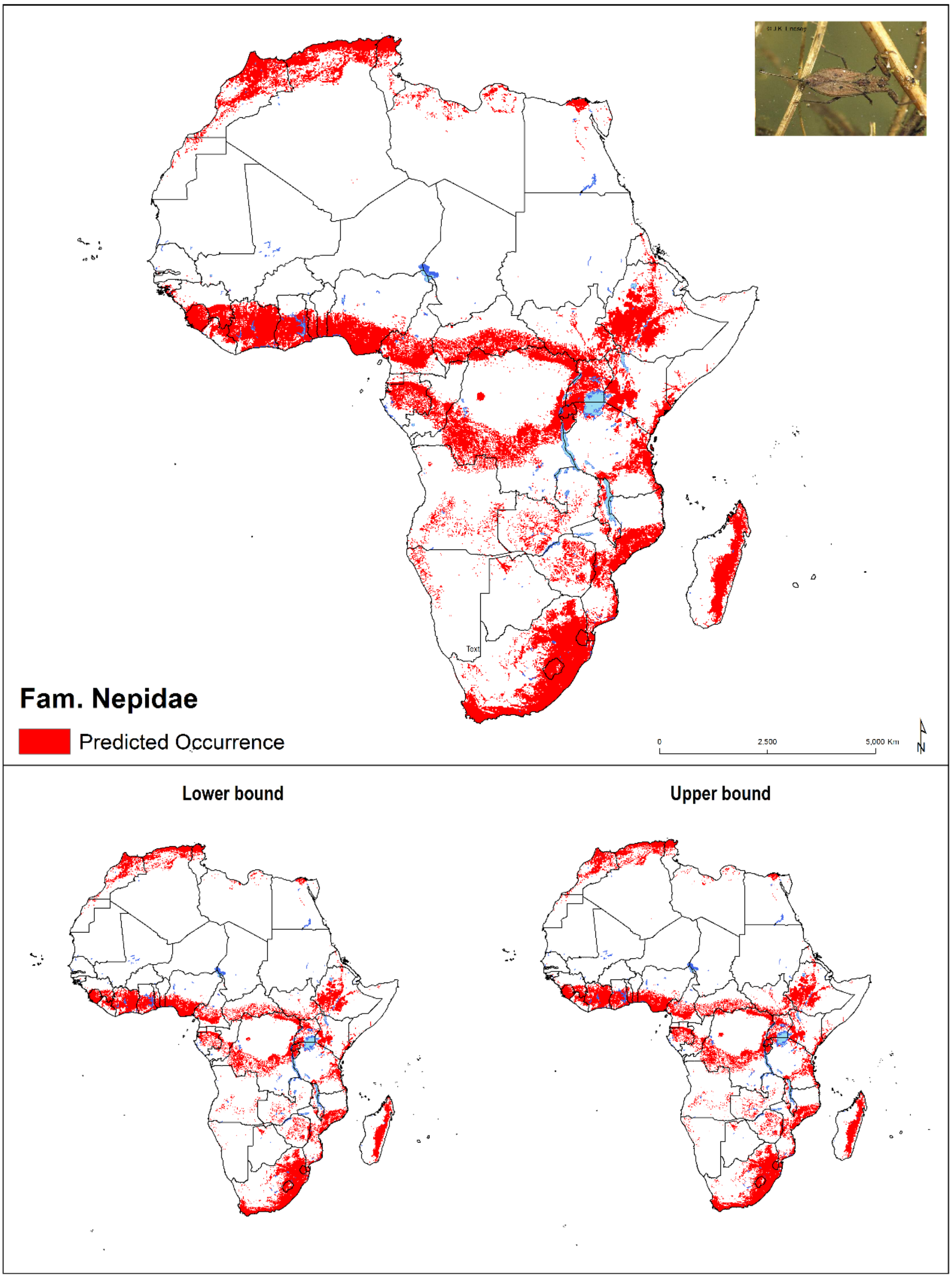

Modelling the spatial distribution of aquatic insects (Order Hemiptera) potentially involved in the transmission of *Mycobacterium ulcerans* in Africa

Jorge Cano, Antonio Rodriguez, Hope Simpson, Earnest Njih, Jose F. Gómez & Rachel L Pullan

Figure S15. Partial dependence plots of the relative contribution of covariates to the boosted regression tree (BRT) model for Fam. Nepidae, averaged over 80 ensembles. Blue lines represent the mean partial dependence over all 80 BRT ensembles and grey envelopes the standard deviation from the mean. The *y*-axis is the transformed logit response and *x*-axis is the full range of covariates values.

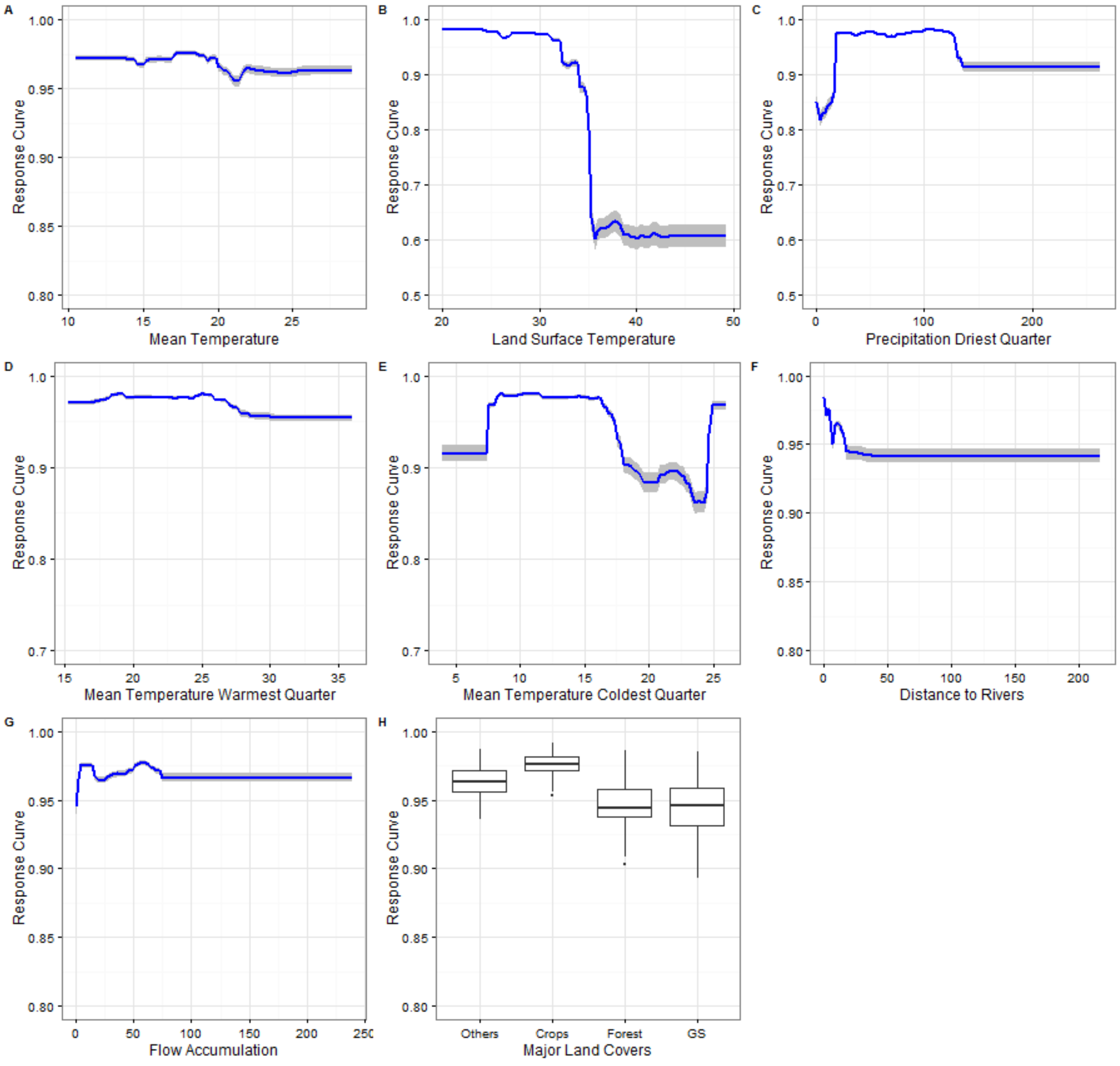

Modelling the spatial distribution of aquatic insects (Order Hemiptera) potentially involved in the transmission of *Mycobacterium ulcerans* in Africa

Jorge Cano, Antonio Rodriguez, Hope Simpson, Earnest Njih, Jose F. Gómez & Rachel L Pullan

Figure S16. Partial dependence plots of the relative contribution of covariates to the random forest (RF) model for Fam. Nepidae, averaged over 80 ensembles. Blue lines represent the mean partial dependence over all 80 RF ensembles and grey envelopes the standard deviation from the mean. The *y*-axis is the transformed logit response and *x*-axis is the full range of covariates values.

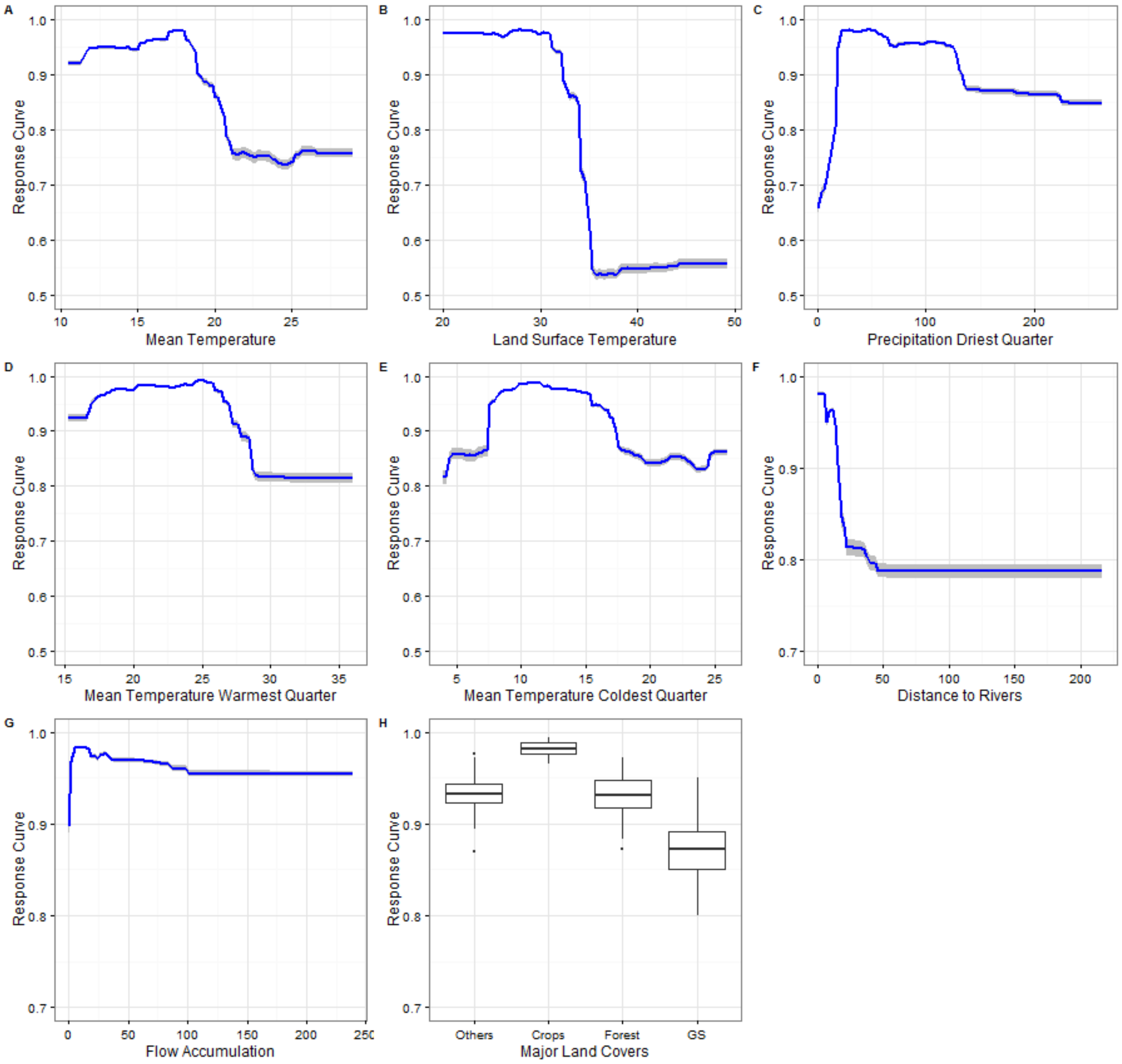

Modelling the spatial distribution of aquatic insects (Order Hemiptera) potentially involved in the transmission of *Mycobacterium ulcerans* in Africa

Jorge Cano, Antonio Rodriguez, Hope Simpson, Earnest Njih, Jose F. Gómez & Rachel L Pullan

Text S4. Description of ecological niche for the Hemiptera insects of the Fam. Nepidae across Africa.

Nepidae species distribution is restricted to coastal areas of western and eastern Africa, northern Africa and eastern areas of South Africa. Our model depicts a more restricted distribution in middle Africa throughout a double belt, which encircle the upper basin of Congo River. This Hemiptera family would be absent in arid and semi-arid zones. It is also predicted to be absent in the eastern coast of Madagascar.

According to partial effect plots for environmental covariates, their distribution seems to be strongly driven by precipitation, being more tolerant to wide range of mean temperature and tolerate high LST (suitability declines abruptly from 33°C degrees). They also show affinity for agricultural lands over other type of land covers.

Modelling the spatial distribution of aquatic insects (Order Hemiptera) potentially involved in the transmission of *Mycobacterium ulcerans* in Africa

Jorge Cano, Antonio Rodriguez, Hope Simpson, Earnest Njih, Jose F. Gómez & Rachel L Pullan

Corixidae

Figure S17. Environmental suitability for Fam. Corixidae across Africa and prediction uncertainty (95% confidence interval). *Insect image from Wikimedia Commons*

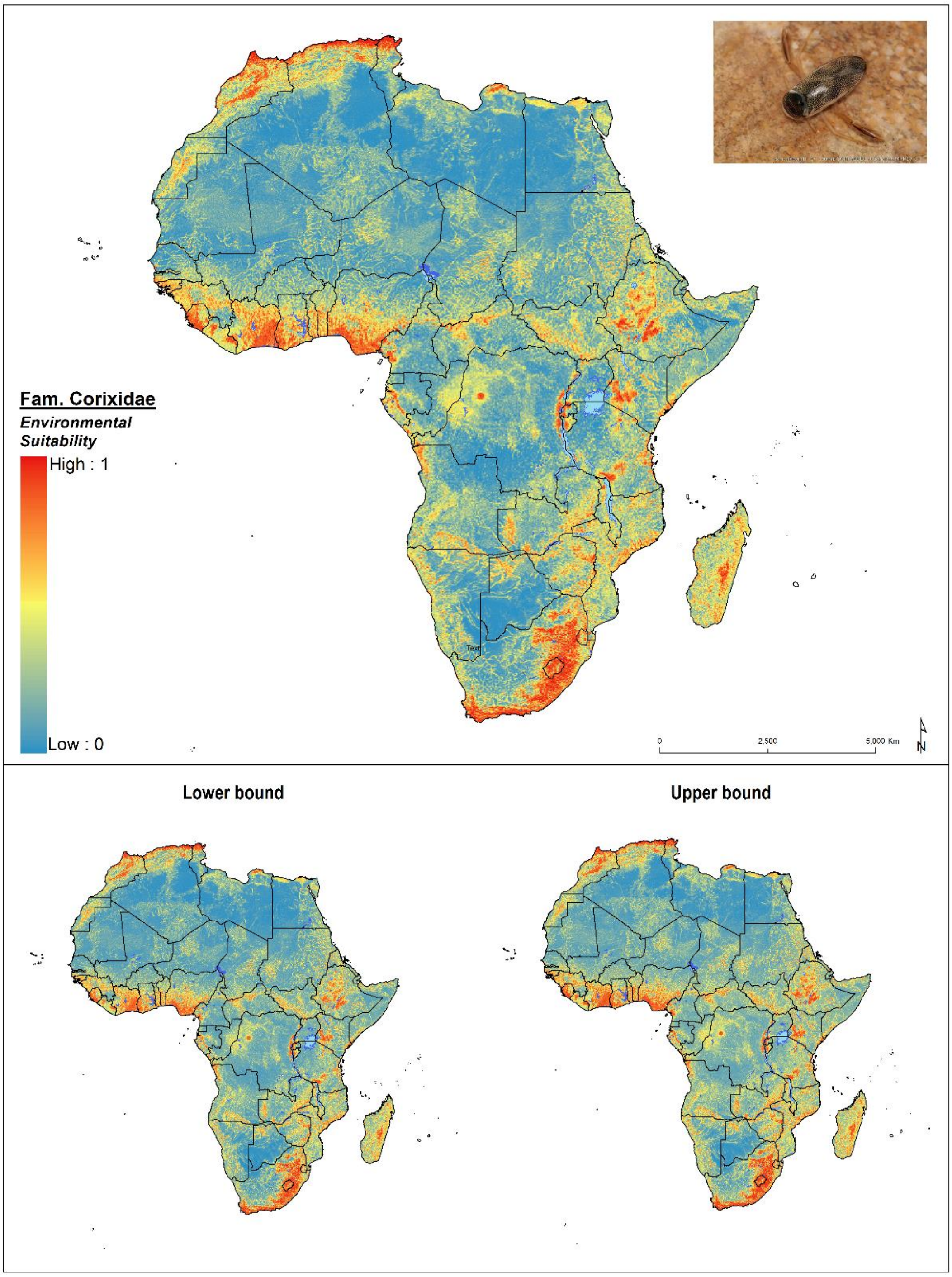

Modelling the spatial distribution of aquatic insects (Order Hemiptera) potentially involved in the transmission of *Mycobacterium ulcerans* in Africa

Jorge Cano, Antonio Rodriguez, Hope Simpson, Earnest Njih, Jose F. Gómez & Rachel L Pullan

Figure S18. Predicted occurrence for Fam. Corixidae across Africa and uncertainty. Optimal threshold was fitted to get better trade-off between sensitivity, specificity and proportion correctly classified (PCC). *Insect image from Wikimedia Commons*

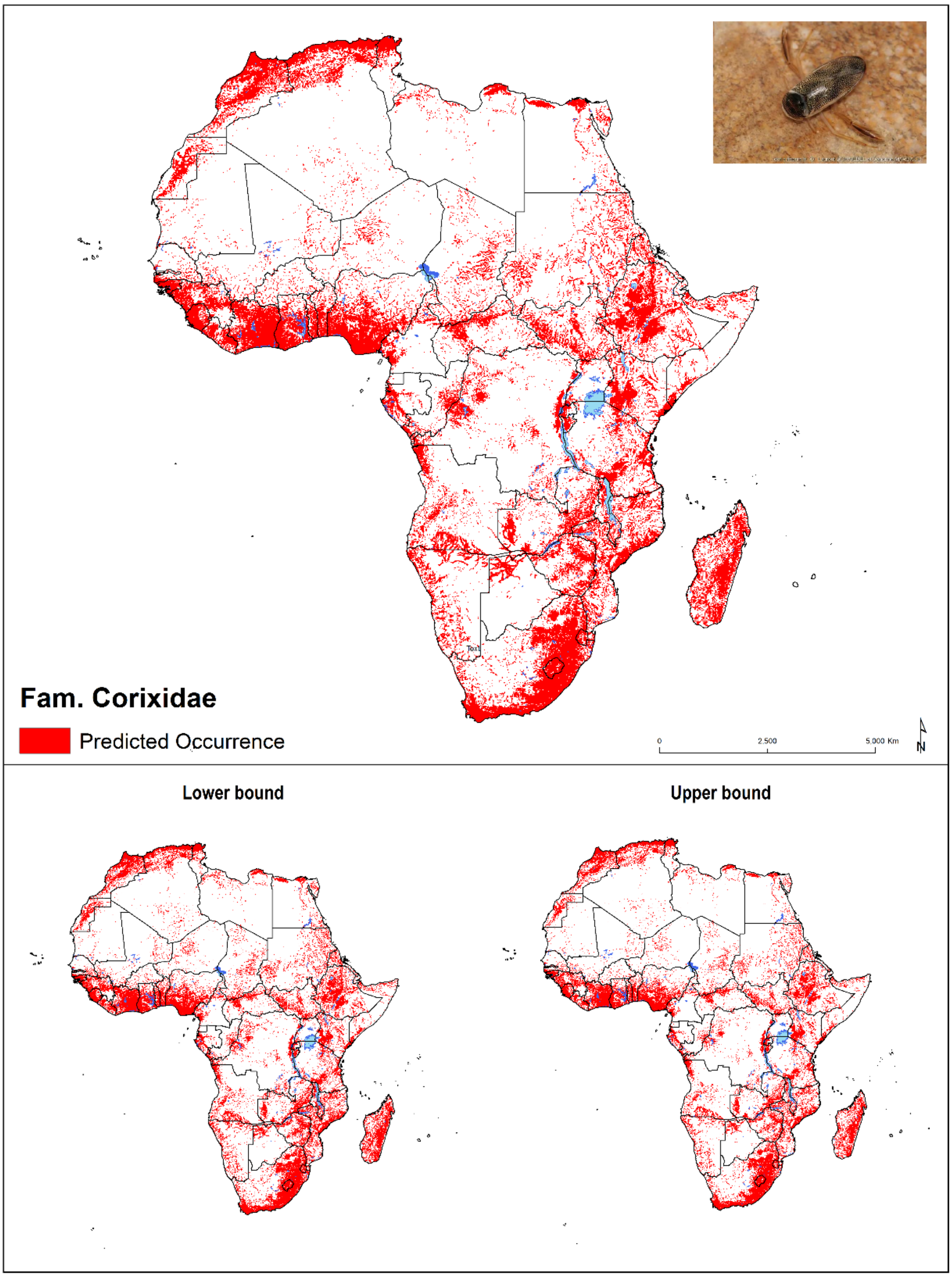

Modelling the spatial distribution of aquatic insects (Order Hemiptera) potentially involved in the transmission of *Mycobacterium ulcerans* in Africa

Jorge Cano, Antonio Rodriguez, Hope Simpson, Earnest Njih, Jose F. Gómez & Rachel L Pullan

Figure S19. Partial dependence plots of the relative contribution of covariates to the boosted regression tree (BRT) model for Fam. Corixidae, averaged over 80 ensembles. Blue lines represent the mean partial dependence over all 80 BRT ensembles and grey envelopes the standard deviation from the mean. The *y*-axis is the transformed logit response and *x*-axis is the full range of covariates values.

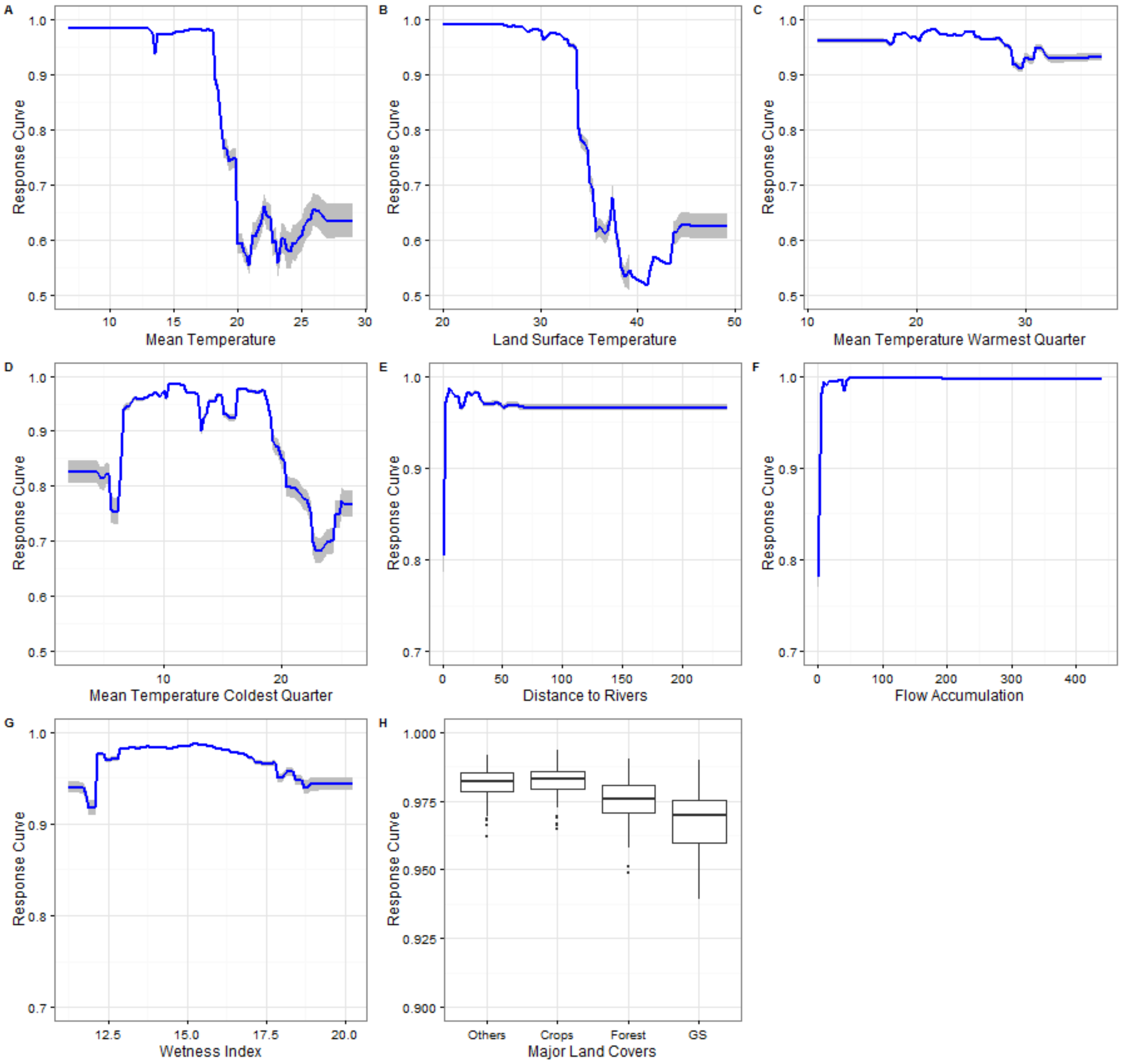

Modelling the spatial distribution of aquatic insects (Order Hemiptera) potentially involved in the transmission of *Mycobacterium ulcerans* in Africa

Jorge Cano, Antonio Rodriguez, Hope Simpson, Earnest Njih, Jose F. Gómez & Rachel L Pullan

Figure S20. Partial dependence plots of the relative contribution of covariates to the random forest (RF) model for Fam. Corixidae, averaged over 80 ensembles. Blue lines represent the mean partial dependence over all 80 RF ensembles and grey envelopes the standard deviation from the mean. The *y*-axis is the transformed logit response and *x*-axis is the full range of covariates values.

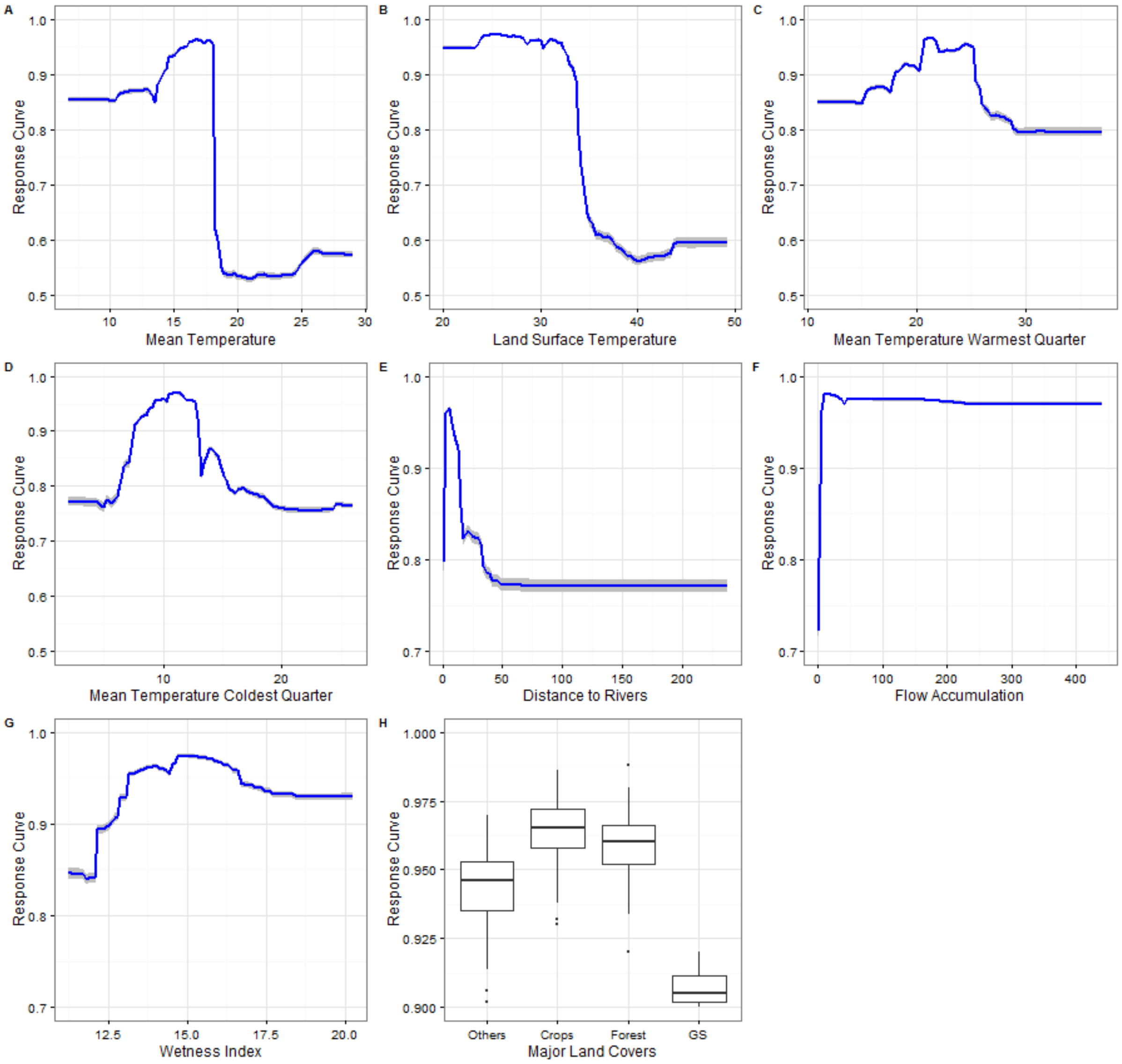

Modelling the spatial distribution of aquatic insects (Order Hemiptera) potentially involved in the transmission of *Mycobacterium ulcerans* in Africa

Jorge Cano, Antonio Rodriguez, Hope Simpson, Earnest Njih, Jose F. Gómez & Rachel L Pullan

Text S5. Description of ecological niche for the Hemiptera insects of the Fam. Corixidae across Africa.

Our ecological model for Fam. Corixidae shows a wide distribution of this Hemiptera family across Africa, even in desert areas at some extent. Nevertheless, it is mostly predicted in wide areas of western and northern Africa, and eastern coast of South Africa. In eastern Africa, this aquatic Hemiptera will also find suitable environments to thrive in both coastal areas and inland. It seems to be nearly absent or scarce in vast areas of middle Africa (i.e. Cameroon, Equatorial Guinea and Democratic Republic of the Congo (DRC). Only coastal areas of Angola, Gabon and Congo offers suitable conditions for this Hemiptera family to prosper across south-west of Africa.

Corixidae species will more likely present in human transformed landscapes, agriculture landscapes according to random forest based models, and forest. The probability of occurrence declines with the distance to rivers and sharply from environment temperatures above 17°C degrees. It appears that species of this Hemiptera family do not tolerate well extreme temperatures, thriving in areas with moderate temperature throughout the year: around 10°C and 20°C at the coldest and warmest months respectively.

Modelling the spatial distribution of aquatic insects (Order Hemiptera) potentially involved in the transmission of *Mycobacterium ulcerans* in Africa

Jorge Cano, Antonio Rodriguez, Hope Simpson, Earnest Njih, Jose F. Gómez & Rachel L Pullan

Gerridae

Figure S21. Environmental suitability for Fam. Gerridae across Africa and prediction uncertainty (95% confidence interval). *Insect image from Wikimedia Commons*

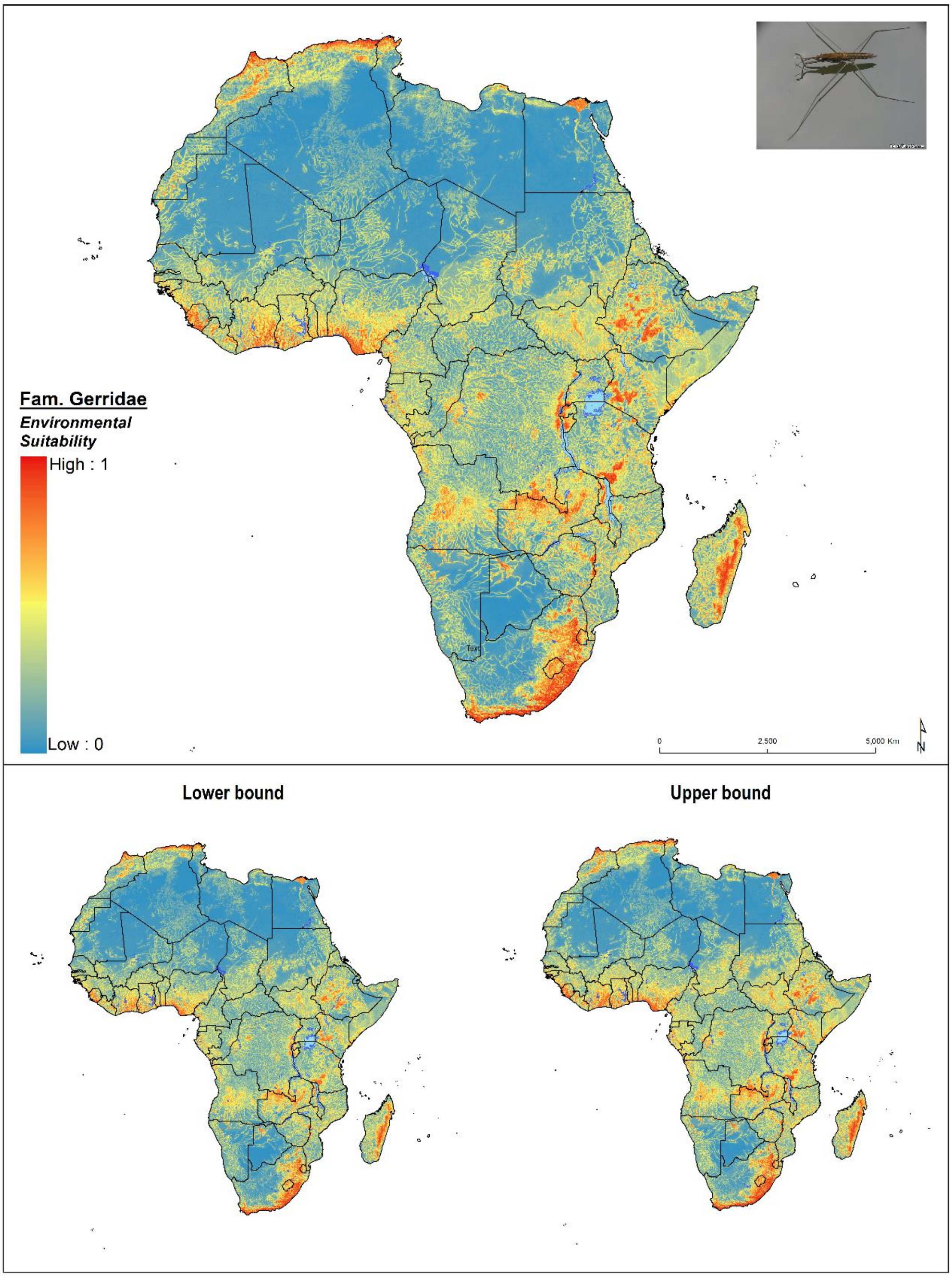

Modelling the spatial distribution of aquatic insects (Order Hemiptera) potentially involved in the transmission of *Mycobacterium ulcerans* in Africa

Jorge Cano, Antonio Rodriguez, Hope Simpson, Earnest Njih, Jose F. Gómez & Rachel L Pullan

Figure S22. Predicted occurrence for Fam. Gerridae across Africa and uncertainty. Optimal threshold was fitted to get better trade-off between sensitivity, specificity and proportion correctly classified (PCC). *Insect image from Wikimedia Commons*

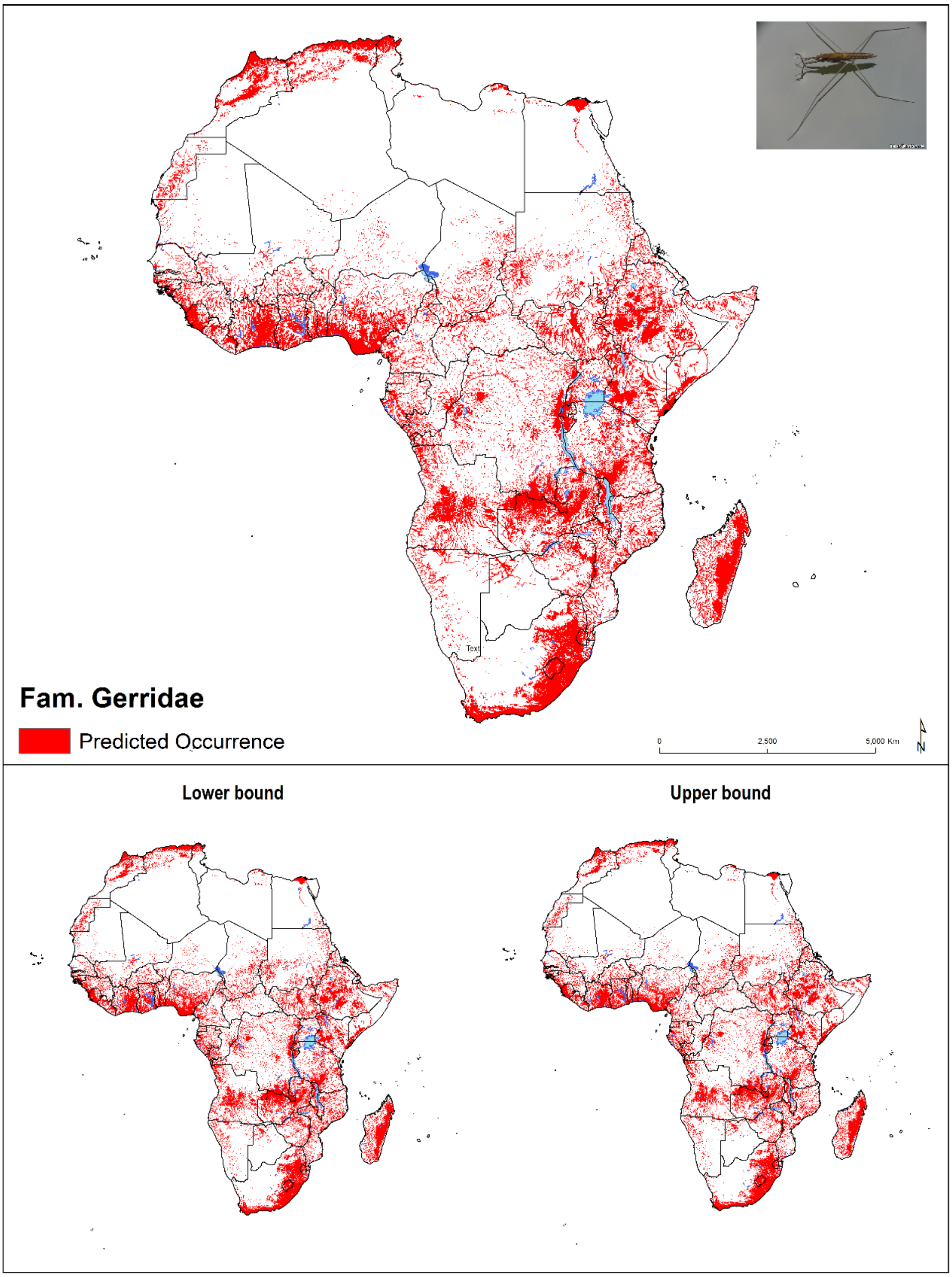

Modelling the spatial distribution of aquatic insects (Order Hemiptera) potentially involved in the transmission of *Mycobacterium ulcerans* in Africa

Jorge Cano, Antonio Rodriguez, Hope Simpson, Earnest Njih, Jose F. Gómez & Rachel L Pullan

Figure S23. Partial dependence plots of the relative contribution of covariates to the boosted regression tree (BRT) model for Fam. Gerridae, averaged over 80 ensembles. Blue lines represent the mean partial dependence over all 80 BRT ensembles and grey envelopes the standard deviation from the mean. The *y*-axis is the transformed logit response and *x*-axis is the full range of covariates values.

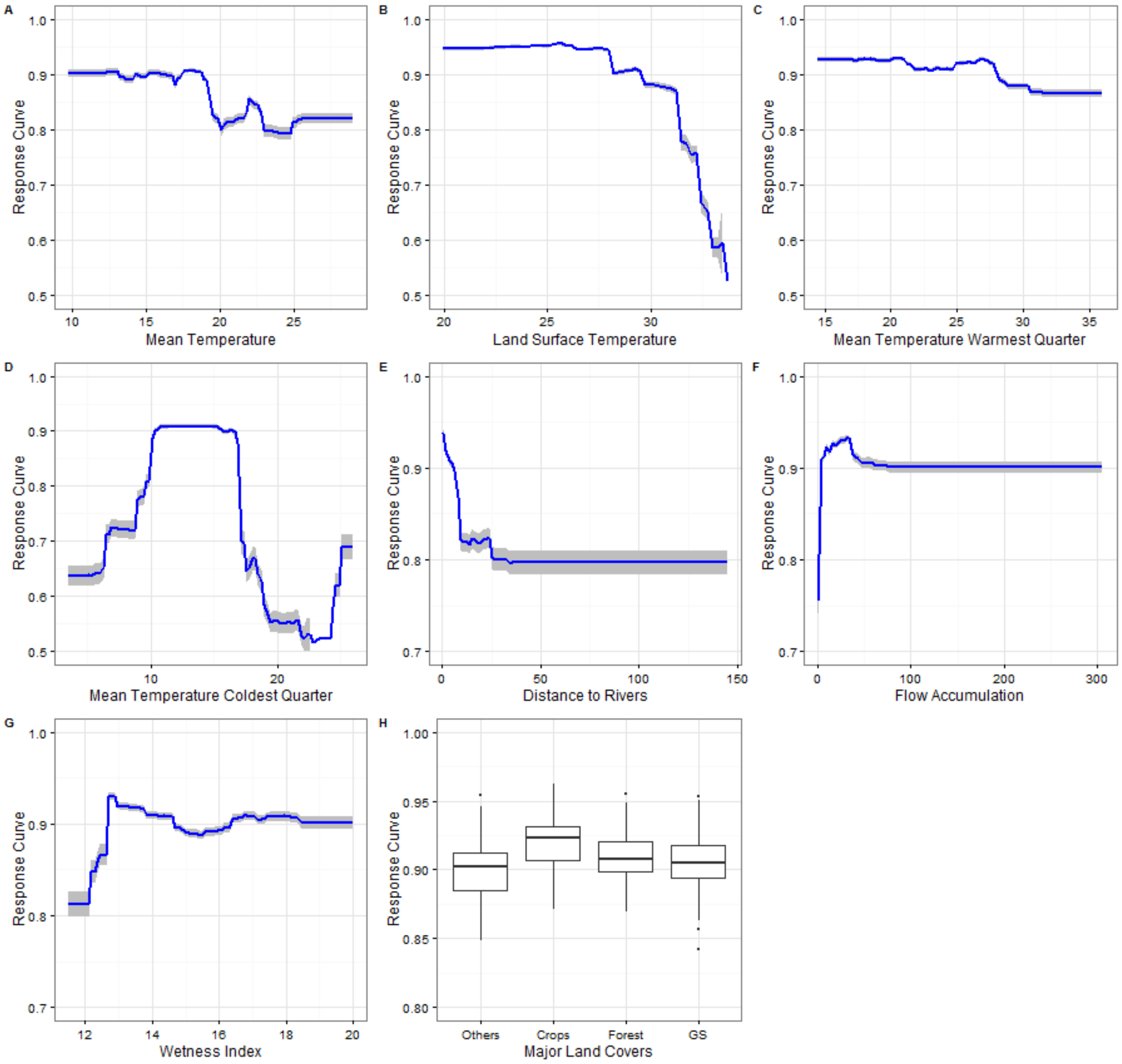

Modelling the spatial distribution of aquatic insects (Order Hemiptera) potentially involved in the transmission of *Mycobacterium ulcerans* in Africa

Jorge Cano, Antonio Rodriguez, Hope Simpson, Earnest Njih, Jose F. Gómez & Rachel L Pullan

Figure S24. Partial dependence plots of the relative contribution of covariates to the random forest (RF) model for Fam. Gerridae, averaged over 80 ensembles. Blue lines represent the mean partial dependence over all 80 RF ensembles and grey envelopes the standard deviation from the mean. The *y*-axis is the transformed logit response and *x*-axis is the full range of covariates values.

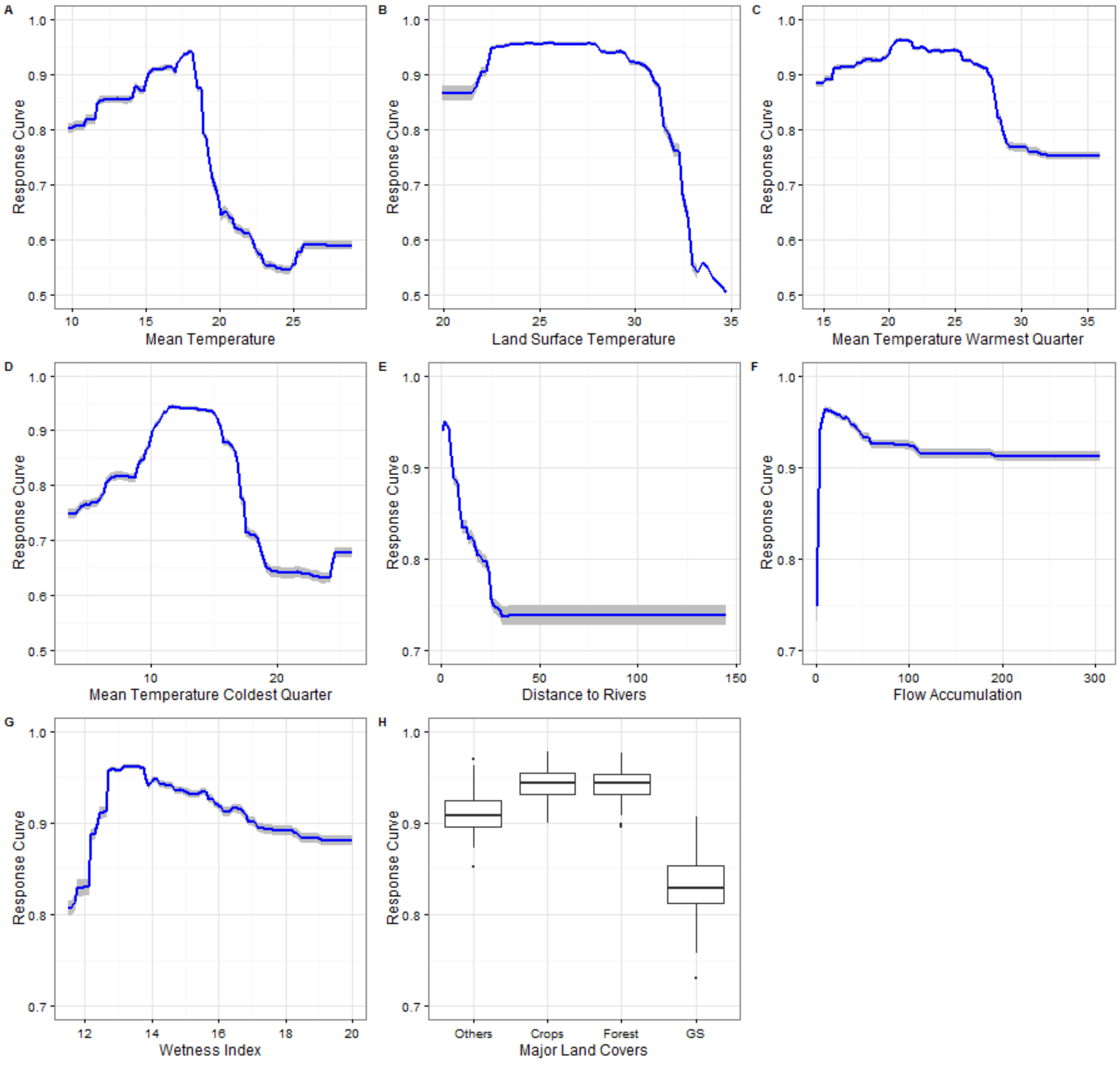

Modelling the spatial distribution of aquatic insects (Order Hemiptera) potentially involved in the transmission of *Mycobacterium ulcerans* in Africa

Jorge Cano, Antonio Rodriguez, Hope Simpson, Earnest Njih, Jose F. Gómez & Rachel L Pullan

Text S6. Description of ecological niche for the Hemiptera insects of the Fam. Gerridae across Africa.

This cosmopolitan Hemiptera family have a more limited distribution in Africa, according to our environmental models. Again, our ensemble model predicts a wide distribution along the western coast and highlands of eastern African countries such as Ethiopia, Tanzania, Great Lakes area and Kenya. Gerridae species are estimated to be present in Southern Africa and in small pockets scattered across middle Africa.

The probability of occurrence declines with the distance to rivers but less sharply comparing to the others Hemiptera families. It seems that agricultural landscapes and forest are more suitable niches for this family, although they are also predicted in urban settings. Less likely to occur in grasslands and shrublands areas. Above 20°C degrees, environment becomes less suitable for species of this Hemiptera family to thrive.
